# NK cell-derived exosomes carry miR-207 and alleviate depression-like symptoms in mice

**DOI:** 10.1186/s12974-020-01787-4

**Published:** 2020-04-22

**Authors:** Dongping Li, Ying Wang, Xinrong Jin, Die Hu, Chunlei Xia, Hanmei Xu, Jialiang Hu

**Affiliations:** 1 The Engineering Research Center of Synthetic Polypeptide Discovery and Evaluation of Jiangsu Province, Zhilan Road 18, Nanjing, 211198 People’s Republic of China; 2 Department of State Key Laboratory of Natural Medicines, China Pharmaceutical University, Nanjing, People’s Republic of China

**Keywords:** Exosomal miR-207, Tril, NK cells, Depression, Inflammatory

## Abstract

**Background:**

Depression is a common mental disease that mainly manifests as bad mood, decreased interest, pessimism, slow thinking, lack of initiative, poor diet and sleep. Patients with severe depression have suicidal tendencies. Exosomes are small vesicles released by the fusion of a multivesicular body and membranes, and they contain specific proteins, nucleic acids, and lipids related to the cells from which they originate. MicroRNAs (miRNAs) are 20*–*24 nt RNAs that can be packaged into exosomes and can play important regulatory roles. Astrocytes are the most abundant cell population in the central nervous system and have a close link to depression. Astrocyte activation could result in the release of inflammatory cytokines, including IL-1β, IL-6, and TNF-α, which could promote the symptoms of depression. In previous research, our team confirmed that NK cells regulate depression in mice. Here, we propose that miRNA in the exosomes from NK cells performs this antidepressant function.

**Methods:**

Exosomes from NK cells were shown by in vivo and in vitro experiments to alleviate symptoms of chronic mild stress in mice and decrease pro-inflammatory cytokines release from astrocytes. The production of pro-inflammatory cytokines was assessed by ELISA. Microarray analysis was used to identify critical miRNAs. Luciferase reporter assays, qPCR, and other experiments were used to prove that exosomal miR-207 has an important role in alleviating the symptoms of stress in mice.

**Results:**

MiRNA-containing exosomes from NK cells could alleviate symptoms of chronic mild stress in mice. In vivo experiments showed that these exosomes decreased the levels of pro-inflammatory cytokines (IL-1β, IL-6, and TNF-α) released by astrocytes. By microarray analysis of exosome miRNA profiles, miR-207 was found to be overexpressed in exosomes derived from unstressed mice. Experiments confirmed that miR-207 directly targets TLR4 interactor with leucine-rich repeats (Tril) and inhibits NF-κB signaling in astrocytes. MiR-207 could decrease the release of pro-inflammatory cytokines and inhibit expression of Tril in vitro. In vivo experiments revealed that exosomes with low miR-207 levels showed decreased antidepressant activity.

**Conclusion:**

Collectively, our findings revealed that exosomal miR-207 alleviated symptoms of depression in stressed mice by targeting Tril to inhibit NF-κB signaling in astrocytes.

## Background

Depression is a mood disorder characterized by indifferent and slow thinking, which includes symptoms of psychomotor retardation and loss of interest in normal things [1]. It is a common and burdensome psychological disorder, and the World Health Organization predicts that it will be the disease with the second highest morbidity in 2020 [2]. It has received widespread attention as a public health priority because its burden causes a risk of suicide and ischemic heart disease [3]. Although the discovery of antidepressants is based on the monoamine hypothesis [4], there has not yet been a highly efficient drug for treating depression.

Some studies have shown that astrocytes are closely related to depression. Astrocytes account for a quarter of the volume of the cerebral cortex [5], which plays roles in homeostasis and defense of the central nervous system (CNS) [6]. Astrocytes are the basis of neural metabolism, homeostasis, behavior, and higher cognitive activity. Quiescent astrocytes provide energy and nutritional support for neurons; regulate synaptic neurotransmission, synaptogenesis, and cerebral blood flow; and maintain the integrity of the blood-brain barrier [7]. Individuals with depression have a decreased number of GFAP^+^ astrocytes in the hippocampus [8]. Furthermore, Sprague-Dawley rats showed depression-like behaviors after receiving L-alpha-aminoadipic acid (an astrocyte-specific toxin) to deplete astrocytes, which confirmed that astrocyte loss can cause depression [9]. Astrocyte activation is the source and target of inflammatory cytokines, including pro-inflammatory cytokines such as interleukin-1-β (IL-1β), interleukin-6 (IL-6), and tumor necrosis factor-α (TNF-α) [10]. IL-1β and TNF-α are important mediators of glial activation and neuronal damage. They also play important roles in regulating neuronal development, neuronal excitability, sleep, and neuroendocrine functions [11]. IL-1β and IL-6 are secreted by reactive astrocytes and can induce inflammation [12, 13], and they were proven to be key factors in inducing depression [14, 15]. TNF-α influences many chemical and immune regulatory pathways that are related to and characteristic of depression [16]. Overall, astrocytes play an important role in depression; however, there are no antidepressant drugs that have been designed to target astrocytes.

TLR4 interactor with leucine-rich repeats (Tril) was initially characterized as a novel component of the TLR4 signaling pathway. Tril is highly expressed in astrocytes and can activate transcription factors such as NF-κB, thereby inducing the production of pro-inflammatory cytokines [17]. This is an important pathway for astrocyte activation.

Exosomes are particles of approximately 50–150 nm in diameter [18] that exist in the blood and other body fluids. They are released from many types of tissues. Within exosomes, there are many proteins, mRNAs and miRNA species [19]. MiRNAs are small noncoding RNAs that regulate mRNA expression and translation [20]. Usually, a miRNA sequence is complementary to a sequence in the 3′UTR of a target gene [21]. MiRNA-mRNA binding leads to the formation of the RNA-induced silencing complex, which sequesters and degrades mRNA to reduce targeting protein expression [22]. In addition, miRNAs exist in exosomes [23]. Therefore, microRNA-containing exosomes often have biological functions that are similar to those of the original cells [24]. In previous studies, we injected NK cells into mice through the tail vein and found that NK cells could improve behavioral tests, which showed that NK cells could alleviate depression-like symptoms in mice by reducing the secretion of inflammatory factors in peripheral blood. Therefore, we wondered if exosomes derived from NK cells could alleviate depression-like symptoms by reducing the secretion of inflammatory factors from astrocytes in the central nervous system.

We showed that NK cell exosomal miRNAs were taken up by astrocytes in vitro and in vivo. This regulated astrocyte activation and reduced the secretion of inflammatory factors to alleviate depression. Finally, we demonstrated that a high level of miR-207 exists in NK cell exosomes, which target Tril to inhibit the NF-κB signaling pathway in astrocytes, which is a molecular mechanism for this antidepressant function.

## Methods

### Animals

Animal experiments were performed in accordance with the guidelines and regulations of the Institutional Animal Care and Use Committee of China Pharmaceutical University. Male BALB/c mice (18–22 g) were purchased from Chang Zhou Cavens Laboratory Animal Center. The mice were raised under specific-pathogen-free conditions for 2–3 weeks to adjust before the experiment.

### Primary cell isolation and culture

#### NK cell isolation and culture

We removed the spleen from the mice and ground it. After suspension in phosphate-buffered solution (PBS), a 200 mesh sieve was used to filter the cells under sterile conditions. The collected cell suspension was centrifuged at 1000 rpm for 5 min. Collected cells were incubated in 3 mL of RBC lysis buffer (Biolegend, San Diego, USA) on ice for 5 min. Then, the cells were washed with PBS. We incubated anti-mouse CD49b-PE (eBioscience, Thermo Fisher Scientific, USA), IgM Isotype Control-PE (eBioscience, Thermo Fisher Scientific, USA), anti-mouse CD3-FITC (eBioscience, Thermo Fisher Scientific, USA), and IgG2b Isotype Control-FITC (eBioscience, Thermo Fisher Scientific, USA) with the cells, and then we sorted CD3^-^CD49b^+^ cells, which represent the NK cell population, by flow cytometry (FACSAria II SORP, BD, USA). The obtained NK cells were cultured in 1640 medium (Invitrogen, USA) containing penicillin (100 U/mL), streptomycin (100 mg/mL), and 10% fetal bovine serum (FBS) (Invitrogen, USA).

#### Astrocyte isolation

The hippocampus was extracted from unstressed and stressed mice. The tissue was digested by treatment with 0.25% trypsin (containing 0.02% EDTA) for 10 min, and DMEM (Invitrogen, USA) with 10% FBS was used to stop digestion. The cells and tissue debris were centrifuged for 5 min at 1000 rpm, and after suspending in PBS, the mixtures were filtered with a 200 mesh sieve. We incubated anti-ACSA-2-PE (Miltenyi Biotec, USA) and IgG2b Isotype Control-PE (Miltenyi Biotec, USA) with the cells and sorted the astrocyte population by flow cytometry (FACSAria II SORP, BD, USA). These cells were used to detect the release of inflammatory factors such as IL-1β, IL-6, and TNF-α from stressed mice.

#### Primary astrocyte isolation

We isolated neonatal mouse astrocytes for in vitro experiments because of the restrictions related to culturing adult mouse astrocytes. Specifically, brain tissue was extracted from neonatal mice, and the brain tissue was minced and digested with 0.05% trypsin. DMEM (Invitrogen, USA) with 10% FBS was used to stop digestion, and the cells were centrifuged for 5 min at 1000 rpm. Collected cells were cultured in DMEM (Invitrogen, USA) containing penicillin (100 U/mL), streptomycin (100 mg/mL), and 10% fetal bovine serum (FBS) (Invitrogen, California, USA). After culturing the cells for 3 days, suspended cells (mixed glial cells) were discarded. Adherent cells were washed three times with PBS and then were digested with 0.25% trypsin. A complete culture medium was used to stop digestion, and the cells were centrifuged at 1000 rpm for 5 min. Collected cells were cultured in complete medium. After being allowed to grow under stable conditions for 1 day, the adherent cells were used for subsequent experiments.

### Collection and characterization of exosomes

NK cells were cultured in 1640 medium with 10% serum that was depleted of exosomes by centrifugation at 140,000*g* for 9 h. After 48 h of culturing NK cells in this exosome-depleted medium, the medium supernatant was collected and centrifuged at 1500*g* for 15 min, and an exoEasy Maxi kit (Qiagen Bioinformatics, Germany) was used to collect exosomes. Exosomes were observed by transmission electron microscopy (HT7700, Hitachi High-technologies, Japan) and were quantified by qNano Gold (Izon Science, New Zealand). The exosome protein markers CD81 and CD63 were identified by Western blot analysis.

### Exosome tracing in vivo and in vitro

#### Exosome tracing in vivo

Exosomes were obtained as described above, and then they were stained using a PKH26 fluorescent cell linker kit (Sigma-Aldrich, USA) before being intravenously injected into mice. After 12 h and 24 h, the red fluorescence signal was measured by an IVIS Spectrum system (PerkinElmer, USA). The hippocampus, liver, spleen, and kidney were surgically separated, and their fluorescence signals were measured by the IVIS Spectrum. The hippocampus was ground to make a cell suspension, and the cells were observed with a fluorescence microscope (U-LH100HG, Olympus Corporation, Japan). The staining group was injected with PKH26.

#### Exosome tracing in vitro

Exosomes were stained with PKH26 and were added to the medium of primary astrocytes. After 24 h, fluorescence was observed under a fluorescence microscope (U-LH100HG, Olympus Corporation, Japan).

### Evaluation of the antidepressant activity of exosomes in vivo

#### Chronic mild stress (CMS) establishment and behavioral tests

We used chronic mild stress as our animal model, and we used an experimental procedure that was described previously [25]. Behavioral tests included the open field test (OFT), in which we tested horizontal movement (defined as at least three paws in a square), vertical movements (climbing), and exercise time (the mobile time of each mouse). Behavioral tests also included a force swimming test (FST) (recorded time spent immobile in seconds) and tail suspension test (TST) (immobility was defined as hanging in a downright direction with only small movements). All behavioral tests were recorded for 5 min. The other experimental details were the same as those described previously [25].

### Exosome treatment

Exosomes were obtained from a 48-h culture medium of 10^6^ NK cells that were extracted from stressed mice and unstressed mice. The average protein concentration was measured using an enhanced BCA protein assay kit (Beyotime, China) to be 322.12 μg/mL. Two hundred microliters of exosome solution was given to each mouse, which contained 66.42 μg of exosome protein. The doses of fluoxetine in this study were selected as described in previous studies [26, 27]. The experimental arrangement details are shown in Table 1. Behavioral testing was performed after a week of exosome administration.

**Table 1:** Exosome treatment and group arrangement

Groups	Materials	Modes	Dosage	Frequency	Numbers
Stress	Normal saline	i.v.	200 μL	One time	5
Fluoxetine	Fluoxetine	i.g.	10 mg/kg	Three times/week	5
Exo (unstress)	Exosomes from NK cells of unstressed mice	i.v.	66.42 μg	One time	5
Exo (stress)	Exosomes from NK cells of stressed mice	i.v.	66.42 μg	One time	5
NK (unstress)	NK cells of unstressed mice	i.v.	1 × 10^6^ cells	One time	5
Unstress	Normal saline	i.v.	200 μL	One time	5

#### MiR-207 depletion exosome treatment

After the transfection of NK cells with miR-207 inhibitor (ABM, USA), NK cells were cultured for another 48 h. Exosomes in the culture supernatant were collected as described above. Exosomes (66.42 μg of total protein) were administered to stressed mice via tail vein injection. Other grouping details are shown in Table 2. Behavioral testing was performed after 1 week of administration.

**Table 2:** MiR-207 depletion exosome treatment and group arrangement

Groups	Materials	Modes	Dosage	Frequency	Numbers
Stress	Normal saline	i.v.	200 μL	One time	5
Exo (inhibitor NC)	Exosomes from NK cells transfected with negative control inhibitor	i.v.	66.42 μg	One time	5
Exo (miR-207 inhibitor)	Exosomes from NK cells transfected with miR-207 inhibitor	i.v.	66.42 μg	One time	5
Unstress	Normal saline	i.v.	200 μL	One time	5

#### Evaluation of the antidepressant activity of miR-207 in vivo

To reduce harm to experimental mice and to generate minimal effects on behavioral testing of mice, 5 μL of miR-207-agomir (GenePharma, China) and 5 μL of a transfection reagent named GP-siRNA-Mate plus (GenePharma, China) were injected into the midline of the skull with a microsyringe using a 3-mm syringe needle after mice were anesthetized. The needle penetrated the brain by 2 mm so that the hippocampus could absorb miR-207. After 48 h, the behavior of the mice was tested. Other grouping details are shown in Table 3.

**Table 3:** Evaluation of the antidepressant activity of miR-207 in vivo

Groups	Materials	Modes	Dosage	Frequency	Numbers
Stress	Normal saline	i.v.	200 μL	One time	6
miR-207 agomir	miR-207 agomir	Intracranial injection	10 μL	One time	6
Exosomes	Exosomes from unstressed mice	i.v.	66.42 μg	One time	6
Unstress	Normal saline	i.v.	200 μL	One time	6

#### Exosome treatment in vitro

Extracted adult mouse astrocytes were cultured for 24 h, and the release of inflammatory factors was measured. In details, 7 μg of exosomes (from unstressed or stressed mice) was added to the astrocyte medium. After 24 h, the supernatant from the medium was collected, and the concentration of inflammatory factors was detected.

### MiRNA array analysis

MiRNA array profiling was performed with exosomes (from unstressed and stressed mice) (*n* = 3) using the Affymetrix miRNA array platform (OE Biotech, Shanghai, China). RNA was extracted using a mirVana™ PARIS™ kit (Ambion, USA) and was quantified by a NanoDrop ND-2000 (Thermo Scientific, USA). The extracted miRNA samples were labeled and hybridized using a miRNA complete Labeling and Hyb kit (Agilent Technologies, USA). An Agilent scanner and Feature Extraction 10.7.1.1 software (Agilent Technologies, USA) were used to obtain the raw microarray data. The genes that were found to be differentially expressed between the two groups were determined by KEGG and GO analysis. Target gene prediction software, GeneSpring 13.1, was used based on TargetScan, PITA and microRNAorg data. The corresponding files were TargetScan_db.txt, PITA_db.txt, and microRNAorg_db.txt. The three databases were compared to extract common genes.

### Luciferase reporter assay

To determine common binding sites between miR-207 and Tril, a luciferase reporter assay was performed by Hanbio Biotechnology (Shanghai, China). 293T cells were transfected with a luciferase construct containing Tril with the wild-type or a mutated version of the binding site. The cells were cotransfected with miR-207 and negative control. After 6 h, the medium was replaced, and after 48 h, the luciferase activities were tested using a Dual Luciferase kit (Promega, USA).

#### Enzyme-linked immunosorbent assay (ELISA)

We detected the concentrations of monoamine (5-HT, norepinephrine (NE), and dopamine (DA)) and inflammatory factors (IL-6, IL-1β, and TNF-α) in brain tissue and inflammatory factors (IL-6, IL-1β, and TNF-α) in the medium of astrocytes by ELISA (Tianjin Anoric Biotechnology, China). In detail, sample and standard reagents were added to coated microwell plates. Biotin incubation and enzyme conjugation liquid incubation were sequentially performed at 37 °C for 60 min. The plate was washed with a washing solution between each incubation step, and then chromogenic reagent was added. The absorbance value was measured by Multiskan™ FC (Thermo Fisher Scientific) at 450 nm. The concentration was calculated based on a standard curve equation.

### Immunofluorescence and immunohistochemical staining

Immunofluorescence and immunohistochemical staining were performed by Wuhan Servicebio Technology (Shanghai, China). Brain tissues were fixed in 4% paraformaldehyde and then were paraffin-embedded. Primary antibodies for immunofluorescence experiments (NeuN, BrdU, and GFAP) were incubated with sections that were prepared from the hippocampal region. Sections were then incubated with appropriate secondary antibodies. Fluorescence was detected using a fluorescence microscope (U-LH100HG, Olympus Corporation, Tokyo, Japan). The primary antibody for immunohistochemical staining was GFAP, and pictures were taken under a light microscope (CKX41SF, Olympus Corporation, Tokyo, Japan). The results were analyzed by ImageJ software.

### MiRNA mimic or inhibitor transfection

Primary astrocytes were stimulated with 100 ng/mL LPS (Beyotime, China) for 24 h. The doses of LPS in this study were selected based on a previous study [28]. Then, miR-207 mimic (GenePharma, China), negative control (NC) (GenePharma, China), or miR-207 inhibitor (ABM, USA) or inhibitor negative control (ABM, USA) was used to transfect primary astrocytes with RNAifectin^TM^ reagent (ABM, USA). After 24 h, the transfection efficiencies were validated by qPCR.

### Western blot analysis

Astrocytes or exosomes from astrocytes (for 1 × 10^6^ cells) that were transfected with miR-207 mimic or miR-207 mimic NC were suspended in 100 μL (for exosomes) or 400 μL (for astrocytes) of SDS lysis buffer (Beyotime, China) containing PMSF (Beyotime, China). Samples were centrifuged at 12,000 rpm for 5 min to extract proteins. After quantification using a BCA, samples (10 μg of total protein each) were separated on a 12% SDS-PAGE by electrophoresis, and then the protein bands were transferred onto a PVDF membrane (Millipore, Massachusetts Institute of Technology, USA). The PVDF membrane was incubated with the following primary antibodies: NF-κB p65 (diluted 1:500) (Wanleibio, China), p-p65 (diluted 1:500) (Wanleibio, China), IKKα (diluted 1:500) (Wanleibio, China), and GAPDH (diluted 1:500) (Goodhere Biotechnology, China). After incubation with the appropriate secondary antibodies (diluted 1:500) (Goodhere Biotechnology, China), the blots were detected by enhanced chemiluminescence reagents (Beyotime, Jiangsu Province, China) and Tanon 5200 Multi imager (Tanon Science & Technology, China).

### MiRNA and mRNA qPCR assays

#### MiRNA qPCR assay

MiRNAs from cells were extracted using an RNAeasy™ small RNA isolation kit with spin columns (Beyotime, China). MiRNAs from exosomes were extracted by an exoRNeasy Serum/Plasma Maxi kit (Qiagen, Germany), and they were reverse transcribed to generate cDNA by a miRNA cDNA synthesis kit with poly(A) polymerase tailing (ABM, USA). miR-207 was detected on a QuantStudio 3 Real-Time PCR system (Applied Biosystems, Thermo Fisher Scientific, USA) using EvaGreen miRNA qPCR mastermix (ABM, USA). miR-207 from exosomes was normalized against the ce-miR-39 expression level, and miR-207 from cells was normalized against the U6 expression level.

#### mRNA qPCR assay

Total RNA was extracted using an RNAeasy™ animal RNA isolation kit with spin columns (Beyotime, China), and then it was reverse transcribed to generate cDNA using 5× ALL-In-One-RT-MasterMix (with AccuRT Genomic DNA Removal Kit) (ABM, USA). qPCR was carried out by a QuantStudio 3 Real-Time PCR system (Applied Biosystems, Thermo Fisher Scientific, USA) using EvaGreen 2× qPCR master mix (ABM, USA). The data were normalized against the GAPDH expression level.

All data were analyzed using QuantStudio 3 Real-Time PCR system software. The primers are shown in Table 4.

**Table 4:** Sequences of the primers

Type	Sequence (5′ to 3′)	Manufacturer
miR-207 primer	F 5′-TTTGCTTCTCCTGGCTCTCC-3′ R 5′-TATCCTTGTTCACGAGTCCTTCAC-3′	GenePharma, Shanghai, China
Tril primer	F 5′-CTATGTATGCCGTTGGGGTAGG-3′ F 5′-AGCTTTTCACTTATTTCGCCCAT-3′	General Biosystems, Anhui, China
GAPDH primer	F 5′-CACTGAGCATCTCCCTCACA-3′ R 5′-GATTGAGCCTGCTTCACCTC-3′	General Biosystems, Anhui, China
miR-207 mimic/agomir	Sense 5′-GCUUCUCCUGGCUCUCCUCCCUC-3′ Antisense 5′-GGGAGGAGAGCCAGGAGAAGCUU-3′	GenePharma, Shanghai, China
miR-207 mimic NC	Sense 5′-UUCUCCGAACGUGUCACGUTT-3′ Antisense 5′-ACGUGACACGUUCGGAGAATT-3′	GenePharma, Shanghai, China
miR-207 inhibitor	5′-GAGGGAGGAGAGCCAGGAGAAGC-3′	ABM, USA
miR-207 inhibitor NC	5′-CAGUACUUUUGUGUAGUACAA-3′	ABM, USA
U6	F 5′-CAGCACATATACTAAAATTGGAACG-3′ R 5′-ACGAATTTGCGTGTCATCC-3′	GenePharma, Shanghai, China

### Co-culture assay

After transfection with a Cy3-miR-207 mimic (GenePharma, China), NK cells (10^5^/well) were cocultured with astrocytes using a transwell plate (3 μm) for 48 h. Astrocytes were placed in the lower chamber, and NK cells were placed in the upper chamber. The control group was NK cells treated with Cy3 dye only (without miR-207 mimic). After washing with PBS, the fluorescence in astrocytes was observed using a fluorescence microscope (U-LH100HG, Olympus Corporation, Japan).

### Statistical analysis

All data are expressed as the mean ± SEM. One-way analysis of variance (ANOVA) followed by Newman-Keuls’ test as a post hoc test was used to analyze more than two groups (*P < 0.05, **P < 0.01, and ***P < 0.001). Student’s *t* tests were used to compare two groups (*P < 0.05, **P < 0.01, and ***P < 0.001). Data were processed by using GraphPad Prism 5 software (GraphPad) and SPSS 19.0.

## Results

### Exosomes released from NK cells

**Fig. 1:**
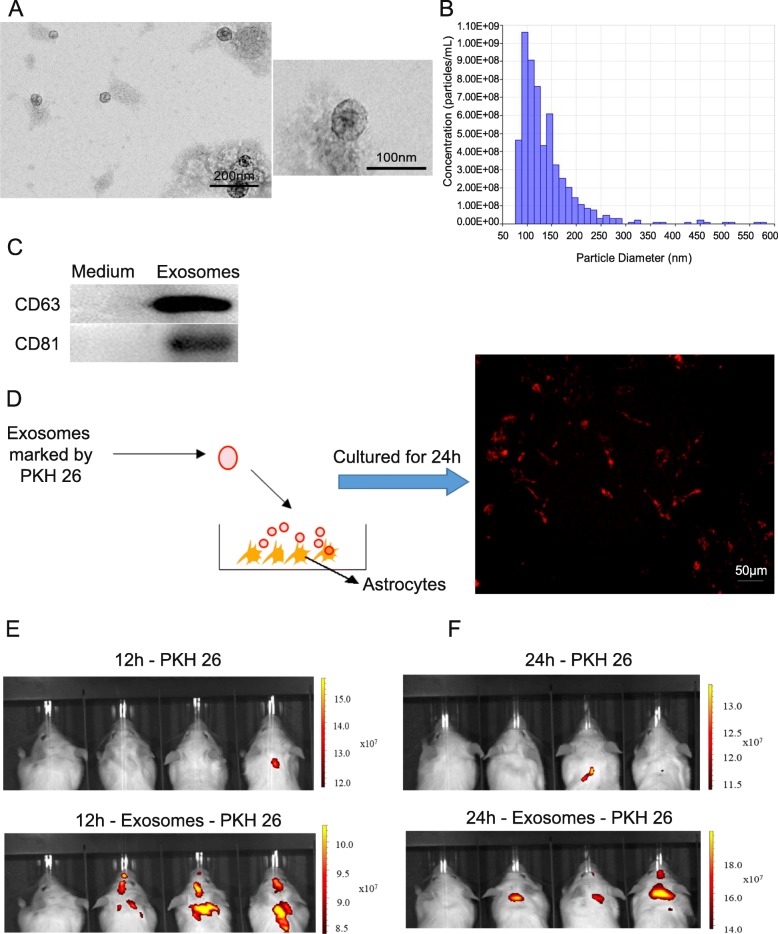
Identification of exosomes isolated from NK cells and the uptake analysis of exosomes by astrocytes. **a** Transmission electronic microscopy (TEM) analysis of the exosomes secreted by NK cells. Scale bars indicate 200 nm and 100 nm, respectively. **b** Measurement of particle size of the vesicles secreted from NK cells by qNano Gold analysis. **c** Western blot analysis of the expression of the exosomal markers CD63 and CD81 in NK cell-derived exosomes. **d** Exosomes from NK cells were labeled with PKH26 and were taken up by astrocytes extracted from neonatal mice for 24 h. The red fluorescence signal in astrocytes was detected with a fluorescence microscope (bar = 50 μm). **e** Fluorescence distribution in the mouse brain was detected 12 h after exosomes that were stained with PKH26 fluorescent dye were intravenously injected into mice. 12h-PKH26 indicates the fluorescence distribution in the brain 12 h after PKH26 fluorescent dye was injected into mice; 12h-exosomes-PKH26 indicates the fluorescence distribution in the brain, and 12 h after exosomes marked by PKH26 dye were injected into mice. The left mouse in both pictures was used as a control mouse; these mice were injected with normal saline without dye molecules. **f** Fluorescence distribution in the brain 24 h after exosomes that were stained with PKH26 fluorescent dye were injected into the tail vein of mice. 24 h-PKH26 indicates the fluorescence distribution in the brain 24 h after PKH26 fluorescent dye was injected into mice; 24h-exosomes-PKH26 indicates the fluorescence distribution in the brain 24 h after exosomes that were marked by PKH26 dye were injected into mice. The left mouse in both pictures was used as a control mouse; these mice were injected with normal saline without dye molecules

We first determined if NK cells could secrete exosomes that could pass through the blood-brain barrier in vivo and be taken up by target cells. We extracted NK cells from mouse spleens by sorting with flow cytometry. After analyzing the whole cell group, the proportion of NK cells (CD49b^+^CD3^−^) in the mouse spleen was found to be 5.69%, and the purity of CD49b^+^CD3^−^ cells obtained was greater than 90% (Figure S1A). We cultured the cells for 48 h and collected the supernatant to isolate exosomes. After isolation and purification from NK cell culture medium, morphological assessment with transmission electron microscopy revealed the typical cup-shaped morphology of exosomes (Fig. 1a). qNano Gold analysis revealed that most exosome particles had a diameter of 50 to 150 nm (Fig. 1b). Western blot analysis confirmed the presence of the exosomal markers CD63 and CD81 (Fig. 1c). Then, we determined whether these exosomes could be taken up by astrocytes. Exosomes were labeled with the fluorescent dye PKH26, and exosomes were added to the culture medium of primary astrocytes. After 24 h, there were red fluorescence staining in these cells, which suggested that astrocytes could uptake the exosomes (Fig. 1 d). To determine whether exosomes could pass through the blood-brain barrier in vivo, we injected exosomes labeled with PKH26 through the mouse tail vein. After 12 h and 24 h, we detected the fluorescence signal in the mouse head (Fig. 1e, f) (the left mouse was a control injected with no dye) in the exosome-PKH26 group, and almost no fluorescence signal was observed in the PKH26 group. These results suggested that exosomes could cross the blood-brain barrier to the brain. Then, we extracted organs to detect the fluorescence signal for further confirm the observed results. We observed a fluorescence signal in the hippocampus, liver, and kidney in the exosome-PKH26 group (Figure S1B-C). We also ground the hippocampus into a cell suspension, and red fluorescence was observed at the cellular level (Figure S1D-E) in the exosome-PKH26 group. Overall, our results showed that NK cells can secrete exosomes, which are transported through the blood-brain barrier in vivo and are taken up by target cells.

### Evaluation of the antidepressant activity of exosomes in vivo

**Fig. 2:**
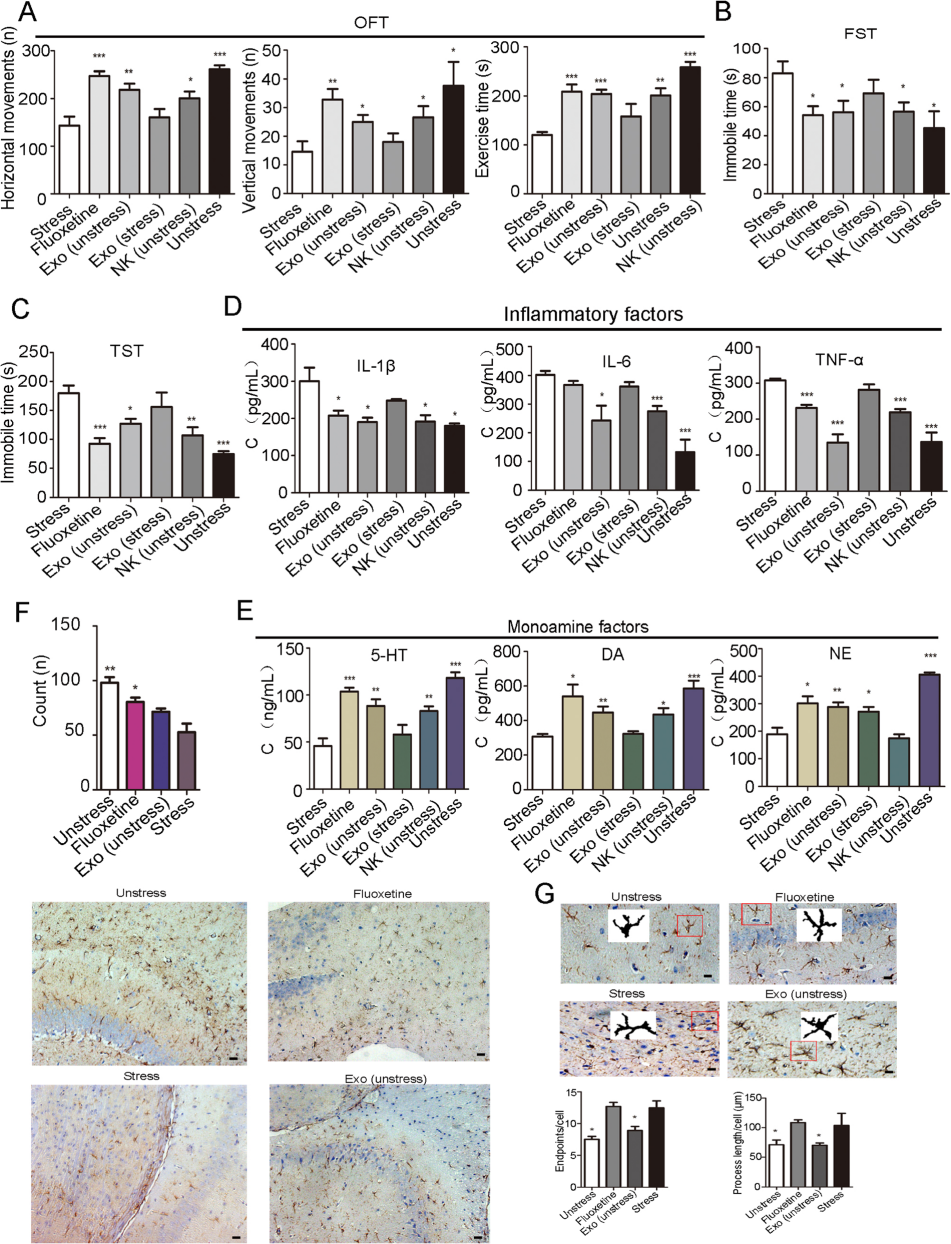
Evaluation of the antidepressant activity of exosomes in vivo. The animal grouping and treatment strategy is shown in Table 1. **a** Behavioral test (OFT), which included horizontal movements (defined as at least three paws in a square), vertical movements (defined as at least three paws in a square) and exercise time (*P < 0.05, **P < 0.01, and ***P < 0.001 compared with the stress group; *n* = 5 mice per group). **b** Behavioral test (FST) (recorded immobile time in seconds) (*P < 0.05 compared with stress group; *n* = 5 mice per group). **c** Behavioral test (TST) (immobility was defined as hanging in a downright direction with only small movements) (*P < 0.05, **P < 0.01, and ***P < 0.001 compared with stress group; *n* = 5 mice per group). **d** The concentrations of inflammatory factors, IL-6, IL-1β, and TNF-α in the mouse brain were detected by ELISA (*P < 0.05 and ***P < 0.001 compared with the stress group; *n* = 5 mice per group). **e** The concentrations of monoamine factors, 5-HT, DA, and NE in the brain were detected by ELISA (*P < 0.05, **P < 0.01, and ***P < 0.001 compared with the stress group; *n* = 5 mice per group). **f** Quantification of GFAP-positive cells in the hippocampus of mice by immunohistochemical analysis. The unstressed group represents the hippocampus of unstressed mice; the fluoxetine group represents the hippocampus of stressed mice that were intravenously injected with fluoxetine; the stress group represents the hippocampus of stressed mice injected with normal saline; and the exo (unstress) group represents the hippocampus of stressed mice injected with exosomes from unstressed mice (*P < 0.05, **P < 0.01, and ***P < 0.001 compared with the stress group; *n* = 5 mice per group; bar = 50 μm). **g** Morphology of astrocytes in the hippocampus of mice analyzed with ImageJ software (*P < 0.05 compared with the stress group; *n* = 5 mice per group; bar = 20 μm)

To evaluate the antidepressant effects of exosomes, we used CMS to establish a mouse stress model. After 30 days, we observed that mice in the stress group had fewer horizontal movements (*P* = 0.0482), fewer vertical movements (*P* = 0.0424), and less exercise time (*P* = 0.0317) in the OFT and had more immobile times in the FST (*P* = 0.0001) and TST (*P* = 0.0310), with significant differences or extremely significant differences (*P < 0.05, ***P < 0.001 Student’s *t* test) when compared with the unstressed group (Figure S2A-C). Furthermore, by immunofluorescence detection, stressed mice revealed decreased numbers of BrdU^+^ cells, GFAP^+^ cells, and NeuN^+^ cells in the hippocampus compared with unstressed mice (*P < 0.05 and **P < 0.01; Student’s *t* test) (Figure S2D-G). The above data showed that the depression model in mice has been successfully established. Subsequently, we performed an animal experiment with six groups of mice, and the drug treatment strategy is shown in Table 1. Behavioral tests showed that mice in the exo (unstress) group, fluoxetine group, NK (unstress) group, and unstress group except the exo (stress) group showed more horizontal movement (*F* (5, 24) = 11.20, *P* < 0.0001), more vertical movement (*F* (5, 24) = 7.693, *P* = 0.0149), more exercise time (*F* (5, 24) = 9.875, *P* < 0.0001) in the OFT, and less immobile time in the FST (*F* (5, 24) = 2.462, *P* = 0.0417) and TST (*F* (5, 24) = 7.881, *P* = 0.0002) than the stress group (Fig. 2 a–c, *P < 0.05, **P < 0.01, and ***P < 0.001; Newman-Keuls’ test). More importantly, inflammatory factors IL-1β (*F* (5, 24) = 6.350, *P* = 0.0007), IL-6 (*F* (5, 24) = 10.76, *P* = 0.0183) and TNF-α (*F* (5, 24) = 18.88, *P* < 0.0001) in mice of the exo (unstress) group, fluoxetine group, NK (unstress) group, and unstressed group except exo (stress) group were decreased compared with that of the stress group (Fig. 2d, *P < 0.05, **P < 0.01, and ***P < 0.001; Newman-Keuls’ test). Monoamine factors 5-HT (*F* (5, 24) = 11.28, *P* < 0.0001), DA (*F* (5, 24) = 7.699, *P* = 0.0002) and NE (*F* (5, 24) = 20.81, *P* < 0.0001) in mice of the exo (unstress) group, fluoxetine group, NK (unstress) group, and unstressed group except exo (stress) group were increased compared with that of the stress group (Fig. 2e, *P < 0.05, **P < 0.01, and ***P < 0.001; Newman-Keuls’ test). According to immunohistochemical analysis and cell morphological analysis, we observed that fluoxetine increased the number of astrocytes (Fig. 2f, *P < 0.05 and **P < 0.01; Newman-Keuls’ test), and exosomes from unstressed mice altered the morphology of astrocytes, which could be the result of inhibiting astrocyte activation (Fig. 2g, *P < 0.05; Newman-Keuls’ test). Overall, the data showed that exosomes from unstressed mice could apparently alleviate symptoms of depression in stressed mice by inhibiting astrocyte activation and the release of inflammatory factors. Exosomes from stressed mice did not have an antidepressant effect.

### In vitro anti-inflammatory activity of exosomes

**Fig. 3:**
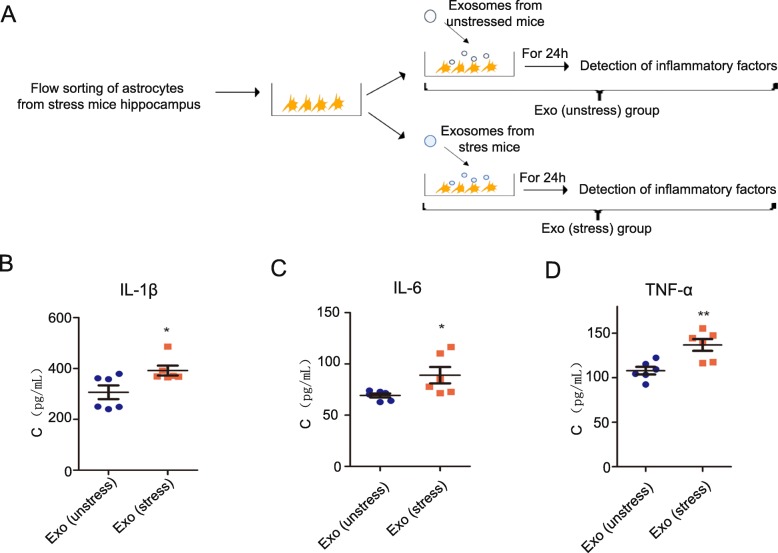
Secretion of inflammatory cytokines from astrocytes derived from stressed mice. **a** Sketch map describes the steps of this experiment, which include astrocyte extraction from the hippocampus of stressed mice by sorting with flow cytometry, NK cell-derived exosome collection from NK cells extracted from unstressed mice and stressed mice, individual inclusion of exosomes with astrocytes, and detection of the concentrations of inflammatory factors released by astrocytes into the medium after a 24-h incubation. **b** The concentration of IL-1β released by astrocytes in the medium (*P < 0.05 compared with the exo (unstress) group; *n* = 6). **c** The concentration of IL-6 released by astrocytes in the medium (*P < 0.05 compared with the exo (unstress) group; *n* = 6). **d** The concentration of TNF-α released by astrocytes in the medium (**P < 0.01 compared with the exo (unstress) group; *n* = 6)

To determine the in vitro effects of exosomes on astrocytes, we isolated astrocytes from the hippocampus of unstressed mice and stressed mice by sorting with flow cytometry. The purities of both populations of astrocytes were in excess of 90% (Figure S3A). The levels IL-1β (*P* < 0.0001), IL-6 (*P* = 0.0001), and TNF-α (*P* = 0.0039) secreted by astrocytes in the cell culture were detected by ELISA, and astrocytes from stressed mice secreted higher levels of these inflammatory cytokines (Figure S3B-D, **P < 0.01 and ***P < 0.001; Student’s *t* test). Furthermore, NK cell-derived exosomes from unstressed mice and stressed mice were separately incubated with astrocytes from stressed mice (Fig. 3a), and after 24 h of incubation, the release of IL-1β, IL-6, and TNF-α in the culture medium was measured. The results showed that exosomes from unstressed mice could decrease the levels of astrocyte-released IL-1β (*P* = 0.0272), IL-6 (*P* = 0.0369), and TNF-α (*P* = 0.0044) (Fig. 3b–d, *P < 0.05 and **P < 0.01; Student’s *t* test).

### MicroRNA expression analysis

**Fig. 4:**
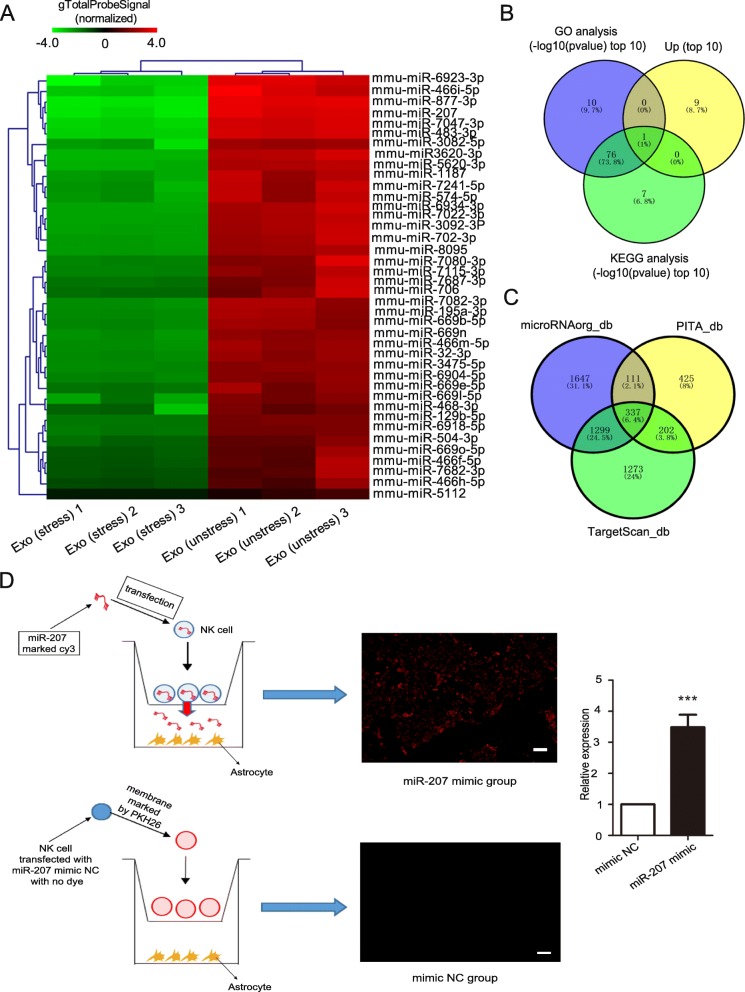
Microarray analysis and exosomal miR-207 uptake by astrocytes. **a** Forty highly expressed miRNAs in NK cell-derived exosomes from unstressed mice compared with those from stressed mice. Data are presented in a heatmap (*n* = 3). **b** Venn diagram of miRNAs of the top 10 GO analyses, top 10 most expressed miRNAs and miRNAs of the top 10 KEGG analyses. **c** Venn diagram of miR-207 target gene analysis using microRNAorg, PITA and TargetScan databases. **d** Exosomal miR-207 was taken up by astrocytes. NK cells transfected with a Cy3’-labeled miR-207 mimic were cocultured with astrocytes in a transwell plate with membrane pores of 3 μm (***P < 0.001 vs. mimic NC group; *n* = 6; bar = 50 μm).

To determine the potential mechanisms underlying the antidepressant activity of exosomes from unstressed mice, we used a miRNA array to analyze the miRNAs in exosomes from unstressed mice (*n* = 3) and stressed mice (*n* = 3) and performed bioinformatics analysis. After differential expression analysis, exosomes from mice in the exo (unstress) group were found to have 40 miRNAs (fold change > 2) that had high expression when compared to their expression in the exo (stress) group (Figure S4A and Fig. 4a). We also performed KEGG pathway enrichment analysis (Figure S4B) and GO analysis, including biological process analysis (Figure S4C), cellular analysis (Figure S4D), and molecular analysis (Figure S4E), for all differentially expressed miRNAs. By these analyses, we identified 87 miRNAs that participated in all top 10 GO analyses (Figure S4F). There was only one miRNA, miR-207, that was included in the top 10 of most highly expressed miRNAs, and in the top 10 of GO analysis and KEGG analysis (Fig. 4b). Target gene prediction analysis (Fig. 4c) showed that 337 potential target genes were identified, and Tril was found to be a key molecule in the activation of astrocytes [17]. Next, we determined whether NK cells could secrete exosome-enclosed miR-207 that could then be transported into astrocytes using an in vitro experiment. After the transfection of a fluorescent Cy3-labeled miR-207 mimic, NK cells were cocultured with astrocytes in a transwell plate (Fig. 4d). The appearance of red fluorescent Cy3 dye in astrocytes demonstrated that the Cy3-miR-207 mimic was delivered from NK cells in the upper transwell to astrocytes in the lower well (Fig. 4d). The miR-207 level was elevated in astrocytes in the miR-207 mimic group. In the mimic NC group, we transfected miR-207 mimic NC and used PKH26 to mark the NK cell membrane, and we found the appearance of red fluorescence in NK cells and no appearance of red fluorescence in astrocytes after a 48-h coculture with NK cells (Fig. 4d). Thus, miR-207 plays an important role in treating stress in mice, and it can be secreted from NK cells and taken up by astrocytes.

### MiR-207 played an important role in astrocyte activation through Tril

**Fig. 5:**
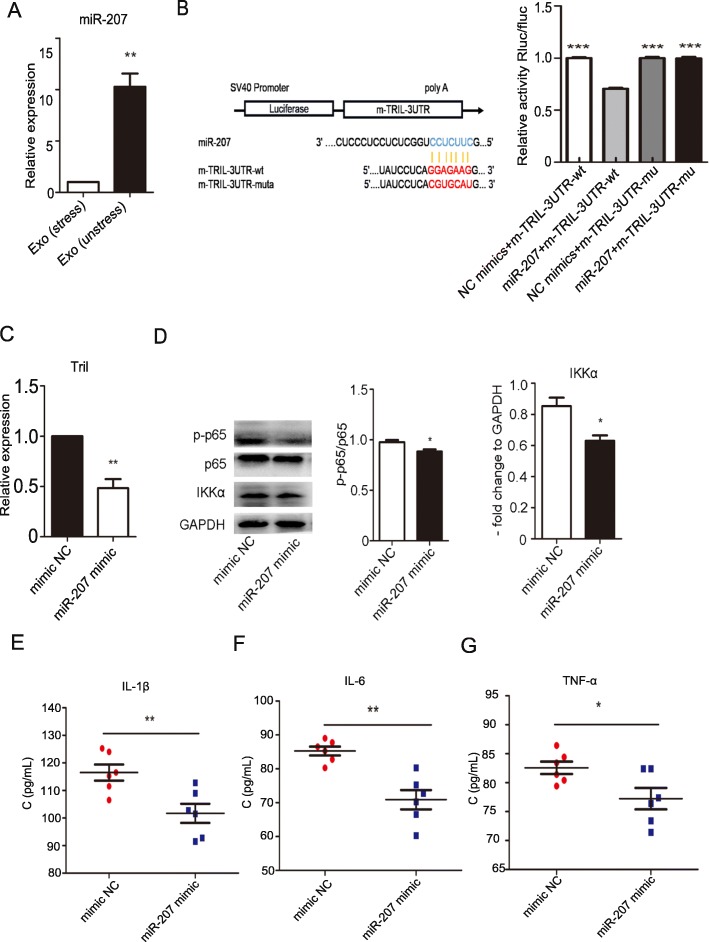
MiR-207 played an important role in astrocyte activation through Tril. **a** The relative expression of miR-207 in exosomes extracted from NK cells of unstressed mice compared with exosomes extracted from NK cells of stressed mice (**P < 0.01 vs. exo (stress) group; *n* = 6). **b** Relative luciferase activity of Tril in the presence of the indicated treatments. Prediction of binding sites between miRNA and Tril and the effects of Tril mutant and wild-type vectors in the presence of miR-207 on fluorescence signal intensity (***P < 0.001 compared with miR-207 and m-TRIL-3UTR-wt group; *n* = 3). (C) qPCR analysis of Tril mRNA expression after transfection of miR-207 mimic in astrocytes (**P < 0.01 compared with the mimic NC group; *n* = 6). **d** Western blot analysis of the protein expression of NF-κB p65, p-p65, IKKα, and GAPDH (*P < 0.05 compared with the mimic NC group; *n* = 3). **e** ELISA analysis of IL-1β concentration after transfection of miR-207 mimic into astrocytes (**P < 0.01 compared with the mimic NC group; *n* = 6). **f** ELISA analysis of IL-6 concentration after transfection of miR-207 mimic into astrocytes (**P < 0.01 compared with the mimic NC group; *n* = 6). **g** ELISA analysis of TNF-α concentration after transfection of miR-207 mimic into astrocytes (*P < 0.05 compared with the mimic NC group; *n* = 6)

First, we confirmed that the miR-207 level was higher in exosomes from unstressed mice than it was in exosomes from stressed mice (Fig. 5a, **P < 0.01; Student’s *t* test). However, it had a lower expression in astrocytes from stressed mice than that from unstressed mice by qPCR analysis (Figure S5A, *P < 0.05; Student’s *t* test). We predicted that the target gene of miR-207 was Tril, which can activate astrocytes. Alignment between the miR-207 sequence and the Tril 3′UTR sequence was performed to show that the Tril coding sequence was a potential target of miR-207 (Fig. 5b). Wild-type and mutated miR-207-binding sites were cloned into luciferase vectors. It was obvious that luciferase activity decreased when Tril was cotransfected with the wild-type binding site vector in the presence of miR-207. However, cells containing the mutated binding site vector did not show such repression (Fig. 5b, ***P < 0.001; Newman-Keuls’ test). Then, we transfected miR-207 mimic into primary astrocytes, which were extracted from neonatal mice and stimulated with LPS (Figure S5B, ***P < 0.001; Student’s *t* test). The level of Tril decreased in miR-207 mimic-treated astrocytes compared with that of mimic NC-treated astrocytes (Fig. 5c, **P < 0.01; Student’s *t* test). We also found that the levels of NF-κB pathway-related molecules, NF-κB p-p65/p65 and IKKα in astrocytes were decreased after transfection with miR-207 (Fig. 5d, *P < 0.05; Student’s *t* test) and the levels of inflammatory factors, IL-1β (*P* = 0.0087), IL-6 (*P* = 0.0010), and TNF-α (*P* = 0.0320), were also decreased (Fig. 5e–g, *P < 0.05 and **P < 0.01; Student’s *t* test). When we transfected astrocytes with miR-207 inhibitor (Figure S5C, **P < 0.01; Student’s *t* test), the levels of Tril (Figure S5D, **P < 0.01; Student’s *t* test) and inflammatory factors such as IL-1β (*P* = 0.0013), IL-6 (*P* = 0.0031), and TNF-α (*P* = 0.0068) were increased (Figure S5E-G, **P < 0.01; Student’s *t* test). These data suggested that miR-207 targeted Tril to inhibit the NK-κB signaling pathway and decrease the release of inflammatory factors.

### In vitro and in vivo antidepressant activity of miR-207-depleted exosomes

**Fig. 6:**
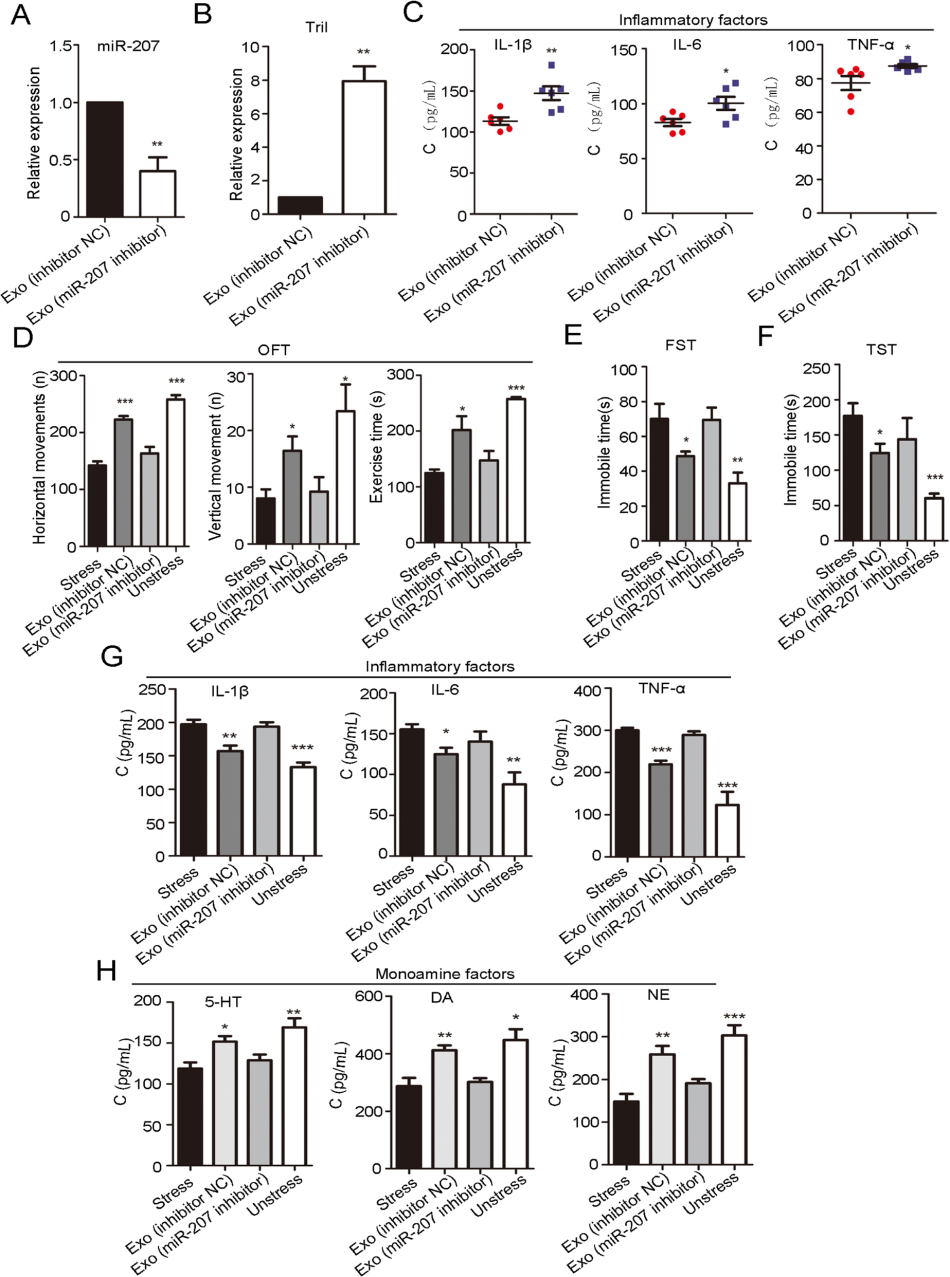
In vitro and vivo antidepressant activity of an exo (miR-207 inhibitor). **a**–**c** The in vitro antidepressant activity of exo (miR-207 inhibitor), transfection of which decreased miR-207 levels in exosomes (*P < 0.05, **P < 0.01 compared with the exo (inhibitor NC) group; *n* = 6). **a** NK cells were transfected with miR-207 inhibitor. After a 48-h incubation, transfected NK cell culture medium was collected for exosome extraction. MiR-207 levels in exosomes were measured and found to be decreased compared with those of the exosomes extracted from NK cells transfected with miR-207 inhibitor NC. **b** Expression of astrocyte Tril after incubation with exosomes with low miR-207 expression were detected by qPCR. **c** Expression of inflammatory factors (IL-1β, IL-6, and TNF-α) by astrocytes after they were incubated with exosomes with low miR-207 expression. **d**–**h** Antidepressant activity of exo (miR-207 inhibitor), which resulted in lower miR-207 levels in exosomes. The animal grouping strategy is shown in Table 2 (*P < 0.05, **P < 0.01, and ***P < 0.001 compared with the stress group; *n* = 5 mice per group). **d** Behavioral test (OFT), which included horizontal movements (defined as at least three paws in a square), vertical movements (defined as at least three paws in a square), and exercise time. **e** Behavioral test (FST) (recorded immobile time in seconds). **f** Behavioral test (TST) (immobility was defined as hanging in a downright direction with only small movements). **g** The concentrations of inflammatory factors, including IL-6, IL-1β, and TNF-α, in mouse brain was measured by ELISA. **h** The concentrations of monoamine factors, 5-HT, DA, and NE, in mouse brain was measured by ELISA

To further determine the key role of exosomal miR-207 in treating stressed mice, we transfected NK cells with miR-207 inhibitor and then isolated exosomes from transfected NK cell culture medium. qPCR analysis confirmed that these exosomes did not contain miR-207 (Fig. 6a, **P < 0.01; Student’s *t* test). When LPS-activated primary astrocytes were incubated with these exosomes, the expression of Tril and inflammatory cytokines, IL-1β (*P* = 0.0053), IL-6 (*P* = 0.0279), and TNF-α (*P* = 0.0417), all increased (Fig. 6b, c, *P < 0.05 and **P < 0.01; Student’s *t* test).

The in vivo activity of miR-207-depleted exosomes was also evaluated. The animal grouping and treatment strategy are shown in Table 2. Behavioral tests showed that mice in the exo (inhibitor NC) group and unstressed group showed more horizontal movement (*F* (3, 16) = 55.64, *P* < 0.0001), more vertical movement (*F* (3, 16) = 5.385, *P* = 0.0094), more exercise time (*F* (3, 16) = 14.60, *P* < 0.0001) in the OFT, and less immobile time in the FST (*F* (3, 16) = 7.501, *P* = 0.0024) and TST (*F* (3, 16) = 6.600, *P* = 0.0041) compared with the stress group; further, there was no significant difference between mice in the exo (miR-207 inhibitor) group and the stress group in the OFT, FST, and TST (Fig. 6d–f, *P < 0.05, **P < 0.01, and ***P < 0.001; Newman-Keuls’ test). Similarly, inflammatory factors, IL-1β (*F* (3, 16) = 17.96, *P* < 0.0001), IL-6 (*F* (3, 16) = 7.228, *P* = 0.0028), and TNF-α (*F* (3, 16) = 22.35, *P* < 0.0001), in mice of the exo (inhibitor NC) group and unstressed group were decreased compared with the levels observed in the stress group; further, there was no significant difference in the levels of inflammatory factors (IL-1β, IL-6, and TNF-α) between mice in the exo (miR-207 inhibitor) group and the stress group (Fig. 6g, *P < 0.05, **P < 0.01, and ***P < 0.001; Newman-Keuls’ test). The monoamine factors 5-HT (*F* (3, 16) = 7.597, *P* = 0.0022), DA (*F* (3, 16) = 9.206, *P* = 0.0009), and NE (*F* (3, 16) = 15.19, *P* < 0.0001) in mice of the exo (inhibitor NC) group and unstressed group were increased compared with the levels in the stress group, and there was no difference in the levels of monoamine factors (5-HT, DA, and NE) between the mice in the exo (miR-207 inhibitor) group and the stress group (Fig. 6h, *P < 0.05, **P < 0.01, and ***P < 0.001; Newman-Keuls’ test).

Furthermore, we injected miR-207-agomir into the mouse brain to evaluate its function in alleviating stress symptoms. First, the efficiency of infection was evaluated by qPCR, and miR-207 levels were found to increase after injections (Figure S6A, **P < 0.01; Student’s *t* test). Next, we used unstressed mice to test the influence of injections on behavioral tests. The results suggested that there were no differences between mice receiving injections in the brain and mice in the control group (Figure S6B-D). Subsequently, we performed an animal experiment with four groups of mice and treated the mice according to the strategy shown in Table 3. Behavioral tests showed that mice in the miR-207-agomir group showed more horizontal movement (*F* (3, 20) = 7.601, *P* = 0.0014), more vertical movements (*F* (3, 20) = 6.073, *P* = 0.0041), more exercise time (*F* (3, 20) = 4.385, *P* = 0.0158) in the OFT, and less immobile time in the FST (*F* (3,20) = 9.288, *P* = 0.0005) and TST (*F* (3, 20) = 8.621, *P* = 0.0007) compared with that of the mice in the stress group (Figure S6E-G, *P < 0.05, **P < 0.01, and ***P < 0.001; Newman-Keuls’ test). Inflammatory factors, IL-1β (*F* (3, 20) = 3.627, *P* = 0.0308), IL-6 (*F* (3, 20) = 7.699, *P* = 0.0013), and TNF-α (*F* (3, 20) = 3.601, *P* = 0.0315), of mice in the miR-207-agomir group decreased compared with that of mice in the stress group (Figure S6H, *P < 0.05, **P < 0.01, and ***P < 0.001; Newman-Keuls’ test). Monoamine factors, 5-HT (*F* (3, 20) = 7.504, *P* = 0.0015), DA (*F* (3, 20) = 7.628, *P* = 0.0014), and NE (*F* (3, 20) = 6.055, *P* = 0.0042), of mice in the miR-207-agomir group increased compared with that of mice in the stress group (Figure S6I, *P < 0.05, and **P < 0.01; Newman-Keuls’ test). The results suggested that exosomal miR-207 was very important in the treatment of stress in mice.

## Discussion

Depression is a common mental disorder with a high incidence, high recurrence rate, and high mortality [29]. At present, the commonly used antidepressants in clinical use increase the concentration of monoamines in the synapse, but these drugs have a slow effect, and for patients with serious symptoms, they typically have no effect [30]. Recent studies have shown that inflammatory cytokines in the brain are involved in an important mechanism of depression [31, 32]. IL-1β and IL-6 have been shown to be increased in some brain regions of patients with depression [33, 34]. Animal studies also showed that exposure to stress could increase IL-1β in several brain areas [35]. TNF-α is another pro-inflammatory cytokine related to the development of depression [36]. Astrocytes can induce inflammatory responses by releasing cytokines such as IL-1β, IL-6, and TNF-α during pathogen stimulation to generate activated astrocytes [14]. Astrocytes are the key factor in depression [37] and could be an important target for treatment. The current study provides several important findings. Firstly, we found that exosomes could improve the behavior of mice, reduce the secretion of inflammatory factors, and increase the release of monoamine factors in the brain. Secondly, exosomes could be absorbed by astrocytes and could reduce the release of inflammatory factors by astrocytes in vivo. Thirdly, exosomal miR-207 may play a potential role against depression by targeting Tril.

Exosomes are membrane-bound nanovesicles and secreted by nearly all cell types in physiological and pathological conditions [38]. In this study, we isolated NK cells from mouse spleens by sorting with flow cytometry, and NK cell-derived exosomes were extracted from the medium after 24 h of culture. After electron microscopy analysis of exosome particle size and Western blot analysis of exosome markers, we injected exosomes labeled with PKH26 through the mouse tail vein, and after 12 h and 24 h, the fluorescence signal was detected in the mouse head. Indeed, exosomes from NK cells could pass through the blood-brain barrier [39] and could carry miRNAs that have regulatory activity by binding target genes [40]. To our knowledge, our study is the first to demonstrate that the miRNAs in exosomes produced by NK cells have antidepressant functions. Exosomal miRNAs can transport epigenetic information, affecting gene expression in a recipient cell [41]. Many studies have demonstrated that exosomal miRNAs can play important roles in treatment or detected markers of many diseases, including cancer progression and metastasis [42, 43], neurodegenerative diseases [44–46], and inflammation [47], and they also participate in the pathological of depression [48, 49]. Furthermore, mesenchymal stem cell-derived exosomal miR26a can improve symptoms of depression in rats [50]. Next, by microarray and bioinformatics analysis, we confirmed that miR-207 had a higher expression in exosomes from unstressed mice compared with those from stressed mice and could be an important role in the treatment of stress mice. A luciferase reporter assay showed that Tril was the target gene. Tril is highly expressed in astrocytes and it is important to activate astrocytes to produce pro-inflammatory cytokines through NF-κB pathway [17]. So we suggested that miR-207 target Tril to inhibit NF-κB signaling in astrocytes to reduce the release of inflammatory factors. To verify this finding, a couple of experiments were done. Firstly, transfecting the miR-207 mimic into astrocytes, we found that the expression of Tril was decreased; the expression of NF-κB pathway-related molecules, NF-κB p-p65/p65 and IKKα, was decreased; and the release of inflammatory factors was decreased. Secondly, transfecting the miR-207 inhibitor into astrocytes, we found that the expression of Tril was increased and the release of inflammatory factors was increased. Thirdly, transfecting the miR-207 inhibitor into NK cells, we found the expression of miR-207 in the exosomes extracted from NK cell culture medium was decreased, and after adding miR-207-depleted exosomes into astrocytes, we found Tril expression in astrocytes was elevated and the release of inflammatory factors was elevated. In vivo experiments showed that the antidepressant effect of exosomes with low miR-207 expression was decreased. Finally, we evaluated the antidepressant effect of miR-207 by a single injection of miR-207 into mice and found that miR-207 and exosomes from unstressed mice had similar antidepressant activity.

## Conclusions

In conclusion, we found that exosomal miR-207 could alleviate the symptoms of stress in mice. A possible mechanism is that after exosomes were intravenously injected into mice, they passed through the blood-brain barrier and were absorbed by astrocytes; then, miR-207 from exosomes entered astrocytes and bound its target gene Tril to decrease its expression, which resulted in decreased activation of NF-κB signaling. Finally, the release of inflammatory factors was decreased, and the stress symptoms in mice were alleviated. The findings of this study provide a new method to treat depression.

## Supplementary information

**Additional file 1:** Figure S1. NK cell isolation and exosome tracing. (A) The isolation and purity of NK cells from stressed mice and unstressed mice. CD49b+CD3- was used to sort cells by flow cytometry. Both groups of cells had a purity in excess of 90%. (B) Fluorescence distribution in the liver, spleen, kidney and hippocampus 12 hours after exosomes stained with PKH26 fluorescent dye were intravenously injected into mice. 12h-PKH26 indicates fluorescence distribution in the liver, spleen, kidney and hippocampus 12 hours after PKH26 fluorescent dye was intravenously injected into mice; 12h-exosomes-PKH26 indicates fluorescence distribution in the liver, spleen, kidney and hippocampus 12 hours after exosomes marked by PKH26 dye were intravenously injected into mice. (C) Fluorescence distribution in the liver, spleen, kidney and hippocampus 24 hours after exosomes stained with PKH26 fluorescent dye were intravenously injected into mice. 24h-PKH26 indicates the fluorescence distribution in the liver, spleen, kidney and hippocampus 24 hours after PKH26 fluorescent dye was intravenously injected into mice; 24h-exosomes-PKH26 indicates the fluorescence distribution in the liver, spleen, kidney and hippocampus 24 hours after exosomes marked by PKH26 dye were intravenously injected into mice. (D) Fluorescence of cells in the hippocampus 12 hours after exosomes stained with PKH26 fluorescent dye were intravenously injected into mice. 12h-PKH26 indicates the fluorescence of cells in hippocampus 12 hours after PKH26 fluorescent dye was intravenously injected into mice; 12h-exosomes-PKH26 indicates the fluorescence of cells in hippocampus 12 hours after exosomes marked by PKH26 dye were injected into mice (Bar=20μm). (E) Fluorescence of cells in the hippocampus 24 hours after exosomes stained with PKH26 fluorescent dye were intravenously injected into mice. 24h-PKH26 indicates the fluorescence of cells in the hippocampus 24 hours after PKH26 fluorescent dye was intravenously injected into mice; 24h-exosomes-PKH26 indicates the fluorescence of cells in the hippocampus 24 hours after exosomes marked by PKH26 dye were intravenously injected into mice (Bar=20μm).

**Additional file 2: **Figure S2. Evaluation of the mouse depression model. (A) Behavioral test (OFT), which included horizontal movements (defined as at least three paws in a square), vertical movements (defined as at least three paws in a square) and exercise time (*P<0.05 compared with the unstressed group. For the unstressed group, *n*=5; for the stress group, *n*=25). (B) Behavioral test (FST) (recorded immobile time in seconds) (***P<0.001 compared with the unstressed group. For the unstressed group, *n*=5; for the stress group, *n*=25). (C) Behavioral test (TST) (immobility was defined as hanging in a downright direction with only small movements) (*P<0.05 compared with the unstressed group. For the unstressed group, *n*=5; for the stress group, *n*=25). (D) Immunofluorescence observation of stressed and unstressed mouse hippocampus for BrdU-positive cells, DAPI-positive cells and their merged signals. (E) Immunofluorescence observation of GFAP-positive cells, DAPI-positive cells and their merged signals in the hippocampus of stressed and unstressed mice. (F) Immunofluorescence identification in stressed and unstressed mouse hippocampus of NeuN-positive cells, DAPI-positive cells and their merged signals. (G) Counting of BrdU-positive, GFAP-positive and NeuN-positive cells (*P<0.05, and **P<0.01 compared with the unstressed group).

**Additional file 3: **Figure S3. Extraction of astrocytes and ELISA analysis of their expression of inflammatory factors. (A) Extraction of astrocytes from stressed mice and unstressed mice by ACSA-2+ sorting with flow cytometry. Both groups of cells had a purity in excess of 90%. (B) IL-1β concentration in astrocyte culture medium (***P<0.001 compared with the unstressed group. *n*=6). (C) IL-6 concentration in astrocyte culture medium (***P<0.001 compared with the unstressed group. *n*=6). (D) TNF-α concentration in astrocyte culture medium (**P<0.01 compared with the unstressed group. *n*=6).

**Additional file 4:** Figure S4. Bioinformatics analysis of miRNA arrays. (A) Heatmap of miRNAs between exo (unstress) exosomes from unstressed mouse NK cells and exo (stress) exosomes from stressed mouse NK cells. (B) Top 20 KEGG pathway analysis of exo (unstress) compared with exo (stress). (C) Top 20 biological process analyses of exo (unstress) compared with exo (stress). (D) Top 20 cellular component analysis of exo (unstress) compared with exo (stress). (E) Top 20 molecular function analyses of exo (unstress) compared with exo (stress). (F) Venn diagram of the top 10 biological process analyses, top 10 cellular component analyses and top 10 molecular function analyses.

**Additional file 5: **Figure S5. Expression of miR-207 in astrocytes and the functions of the miR-207 inhibitor in astrocytes *in vitro*. (A) Relative expression of miR-207 in astrocytes compared with the unstressed group (*P<0.05 compared with the unstressed group. *n*=6). (B) The relative expression of miR-207 in astrocytes after transfection with miR-207 mimic compared with transfection with a mimic NC (***P<0.001 compared with the mimic NC group. *n*=6). (C) The relative expression of miR-207 in astrocytes after transfection with miR-207 inhibitor compared with transfection with an inhibitor NC (**P<0.01 compared with the inhibitor NC group. *n*=6). (D) qPCR analysis of Tril mRNA expression after transfecting astrocytes with miR-207 inhibitor (**P<0.01 compared with the inhibitor NC group. *n*=6). (E) ELISA analysis of IL-1β concentration after transfecting astrocytes with miR-207 inhibitor (**P<0.01 compared with the inhibitor NC group. *n*=6). (F) ELISA analysis of IL-6 concentration after transfecting astrocytes with miR-207 inhibitor (**P<0.01 compared with the inhibitor NC group. *n*=6). (G) ELISA analysis of TNF-α concentration after transfecting astrocytes with miR-207 inhibitor (**P<0.01 compared with the inhibitor NC group. *n*=6).

**Additional file 6: **Figure S6. *In vivo* antidepressant activity of miR-207. (A) Relative expression of miR-207 in the hippocampus after intracranial injection of miR-207 agomir (**P < 0.01 compared with the control group. *n*=4). (B-D) Behavioral test after injection of agomir NC to evaluate the influence of the injection procedure. (B) Behavioral test (OFT), which included horizontal movements (defined as at least three paws in a square), vertical movements (defined as at least three paws in a square) and exercise time, which were compared with the results from the control group. (C) Behavioral test (FST) (recorded immobile time in seconds) results were compared with those of the control group. (D) Behavioral test (TST) (immobility was defined as hanging in a downright direction with only small movements) results were compared with those of the control group. (E-I) *In vivo* antidepressant activity of miR-207. The animal grouping strategy is shown in Table 3. (E) Behavioral (OFT), which included horizontal movements (defined as at least three claws in a square), vertical movements (defined as at least three claws in a square) and exercise time (*P<0.05, **P<0.01 compared with the stress group. *n*=6 in each group). (F) Behavioral test (FST) (recorded immobile time in seconds) (*P<0.05, **P<0.01 compared with the stress group. *n*=6 in each group). (G) Behavioral test (TST) (immobility was defined as hanging in a downright direction with only small movements) (*P<0.05, and ***P<0.001 compared with the stress group. *n*=6 in each group). (H) ELISA analysis of inflammatory factor concentrations, IL-6, IL-1β, and TNF-α, in the brain (*P<0.05, **P<0.01, and ***P<0.001 compared with the stress group. *n*=6 in each group). (I) ELISA analysis of monoamine factor concentrations, 5-HT, DA and NE, in the brain (*P<0.05, **P<0.01, and ***P<0.001 compared with the stress group. *n*=6 in each group).

## Data Availability

All data generated or analyzed during this study were included in this published article and its supplementary files.

## Abbreviations

miRNAs: MicroRNAs
Tril: TLR4 interactor with leucine-rich repeats
CNS: Central nervous system
IL-1β: Interleukin-1-β
IL-6: Interleukin-6
TNF-α: Tumor necrosis factor-α
PBS: Phosphate-buffered solution
FBS: Fetal bovine serum
CMS: Chronic mild stress
OFT: Open field test
FST: Force swimming test
TST: Tail suspension test
ELISA: Enzyme-linked immunosorbent assay
NE: Norepinephrine
DA: Dopamine
NC: Negative control
