# Supplementary figures and images for: NK cell-derived exosomes carry miR-207 and alleviate depression-like symptoms in mice
Source: J Neuroinflammation. 2020 Apr 22;17:126. doi: 10.1186/s12974-020-01787-4 (PMC7178582; doi:10.1186/s12974-020-01787-4)

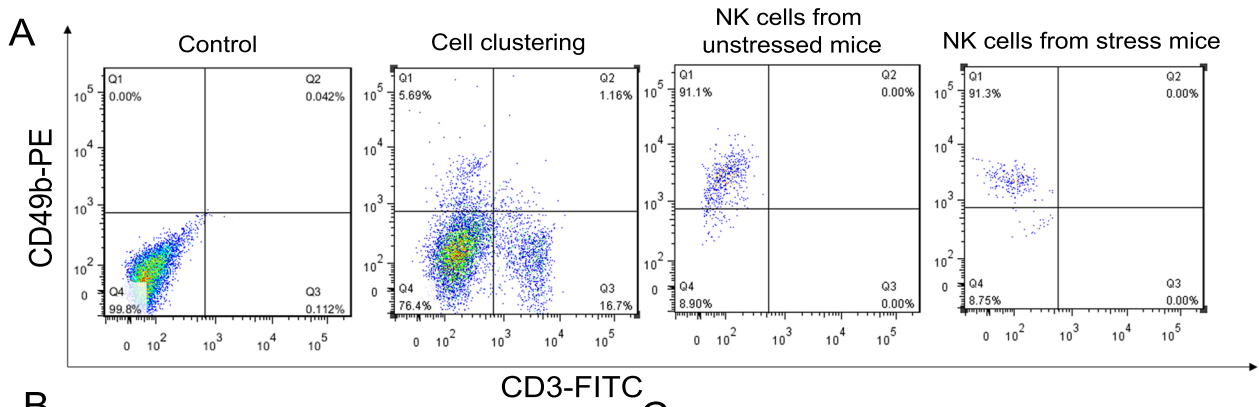

**B**

**C**

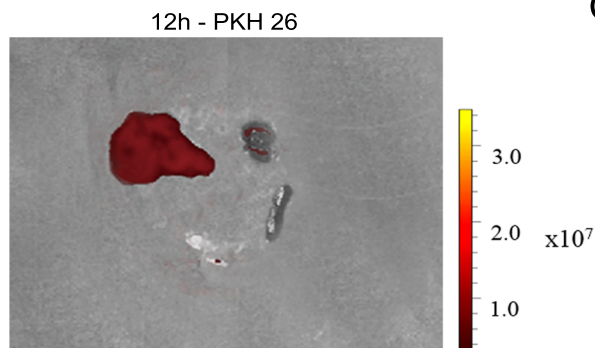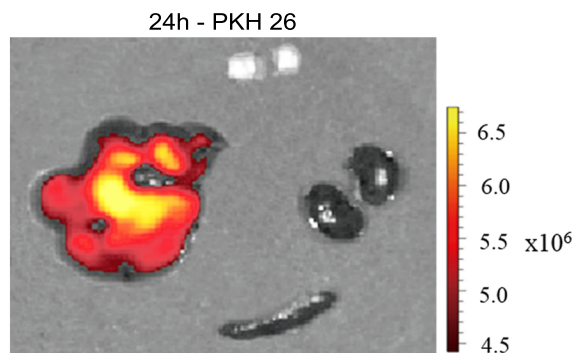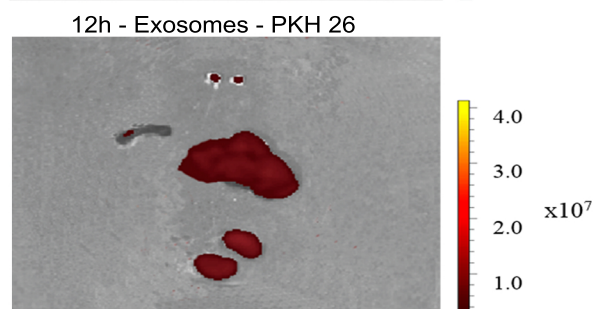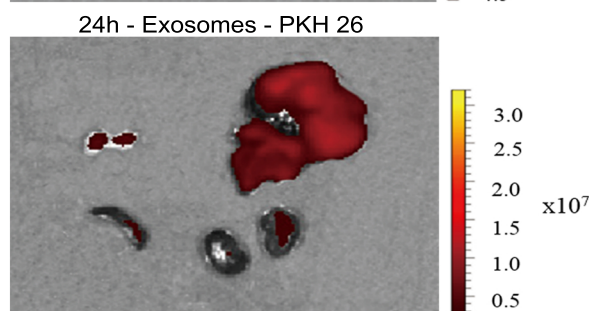

**D**

**E**

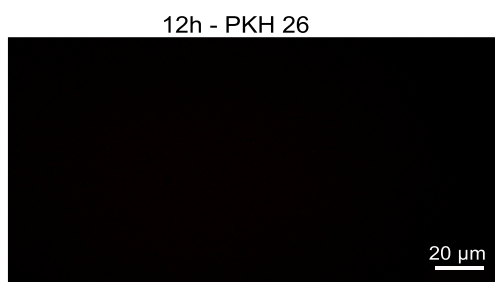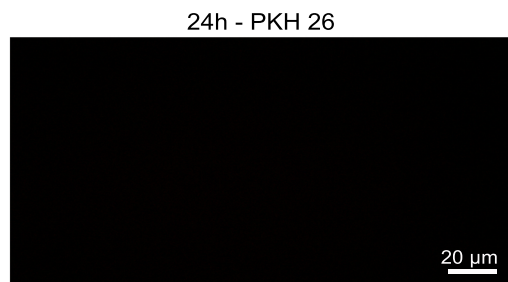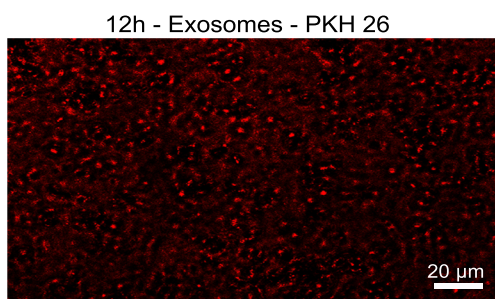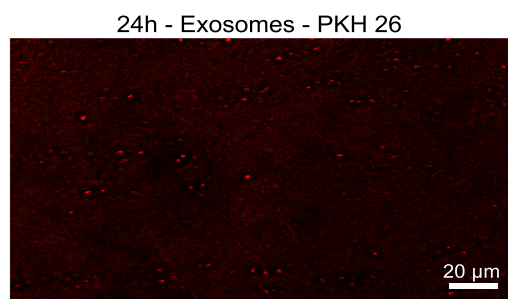

Supplement: Supplementary file 1 — Additional file 1: Figure S1. NK cell isolation and exosome tracing. (A) The isolation and purity of NK cells from stressed mice and unstressed mice. CD49b+CD3- was used to sort cells by flow cytometry. Both groups of cells had a purity in excess of 90%. (B) Fluorescence distribution in the liver, spleen, kidney and hippocampus 12 hours after exosomes stained with PKH26 fluorescent dye were intravenously injected into mice. 12h-PKH26 indicates fluorescence distribution in the liver, spleen, kidney and hippocampus 12 hours after PKH26 fluorescent dye was intravenously injected into mice; 12h-exosomes-PKH26 indicates fluorescence distribution in the liver, spleen, kidney and hippocampus 12 hours after exosomes marked by PKH26 dye were intravenously injected into mice. (C) Fluorescence distribution in the liver, spleen, kidney and hippocampus 24 hours after exosomes stained with PKH26 fluorescent dye were intravenously injected into mice. 24h-PKH26 indicates the fluorescence distribution in the liver, spleen, kidney and hippocampus 24 hours after PKH26 fluorescent dye was intravenously injected into mice; 24h-exosomes-PKH26 indicates the fluorescence distribution in the liver, spleen, kidney and hippocampus 24 hours after exosomes marked by PKH26 dye were intravenously injected into mice. (D) Fluorescence of cells in the hippocampus 12 hours after exosomes stained with PKH26 fluorescent dye were intravenously injected into mice. 12h-PKH26 indicates the fluorescence of cells in hippocampus 12 hours after PKH26 fluorescent dye was intravenously injected into mice; 12h-exosomes-PKH26 indicates the fluorescence of cells in hippocampus 12 hours after exosomes marked by PKH26 dye were injected into mice (Bar=20μm). (E) Fluorescence of cells in the hippocampus 24 hours after exosomes stained with PKH26 fluorescent dye were intravenously injected into mice. 24h-PKH26 indicates the fluorescence of cells in the hippocampus 24 hours after PKH26 fluorescent dye was intravenously i [file 12974_2020_1787_MOESM1_ESM.pdf]

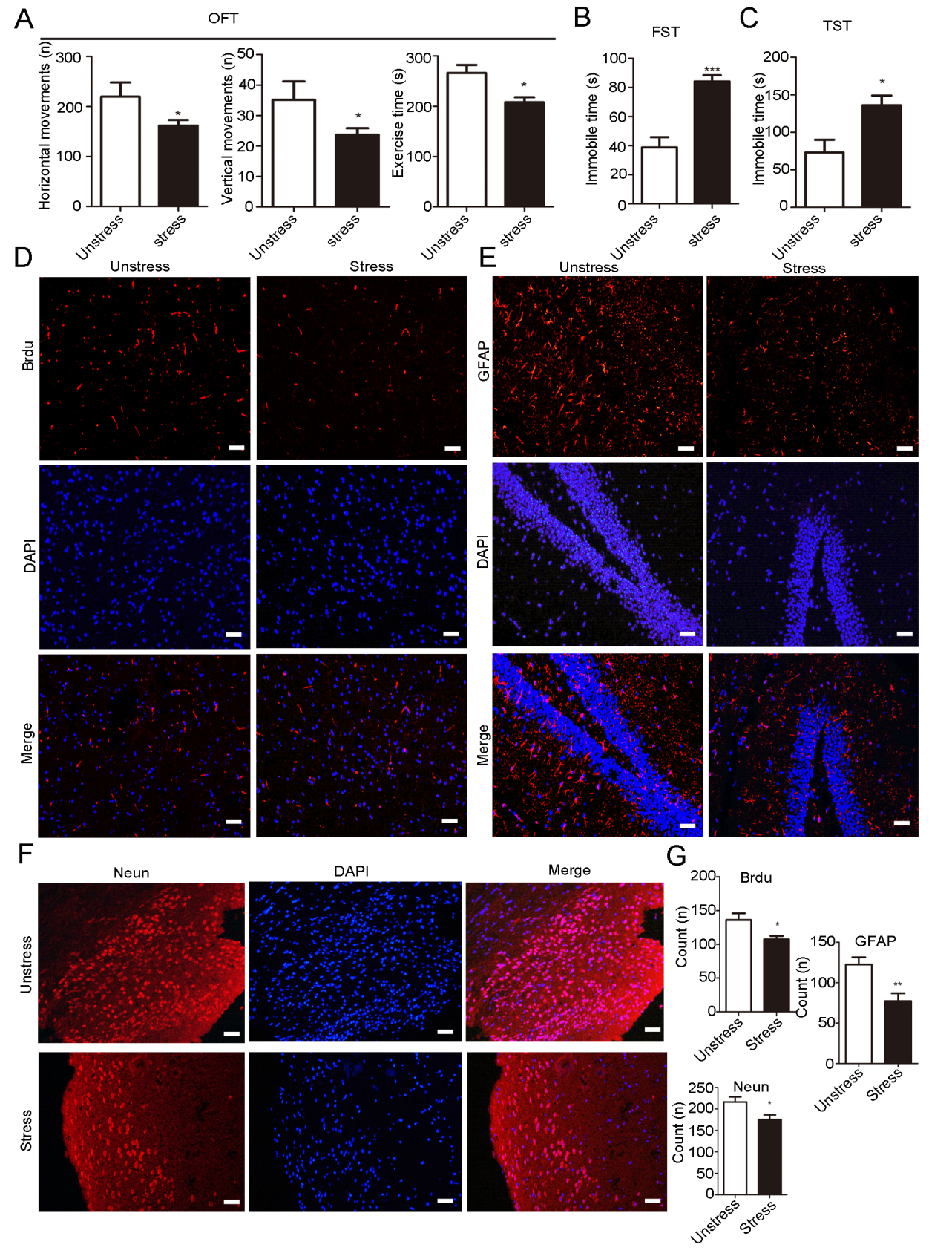

Supplement: Supplementary file 2 — Additional file 2: Figure S2. Evaluation of the mouse depression model. (A) Behavioral test (OFT), which included horizontal movements (defined as at least three paws in a square), vertical movements (defined as at least three paws in a square) and exercise time (*P<0.05 compared with the unstressed group. For the unstressed group, n=5; for the stress group, n=25). (B) Behavioral test (FST) (recorded immobile time in seconds) (***P<0.001 compared with the unstressed group. For the unstressed group, n=5; for the stress group, n=25). (C) Behavioral test (TST) (immobility was defined as hanging in a downright direction with only small movements) (*P<0.05 compared with the unstressed group. For the unstressed group, n=5; for the stress group, n=25). (D) Immunofluorescence observation of stressed and unstressed mouse hippocampus for BrdU-positive cells, DAPI-positive cells and their merged signals. (E) Immunofluorescence observation of GFAP-positive cells, DAPI-positive cells and their merged signals in the hippocampus of stressed and unstressed mice. (F) Immunofluorescence identification in stressed and unstressed mouse hippocampus of NeuN-positive cells, DAPI-positive cells and their merged signals. (G) Counting of BrdU-positive, GFAP-positive and NeuN-positive cells (*P<0.05, and **P<0.01 compared with the unstressed group). [file 12974_2020_1787_MOESM2_ESM.pdf]

A

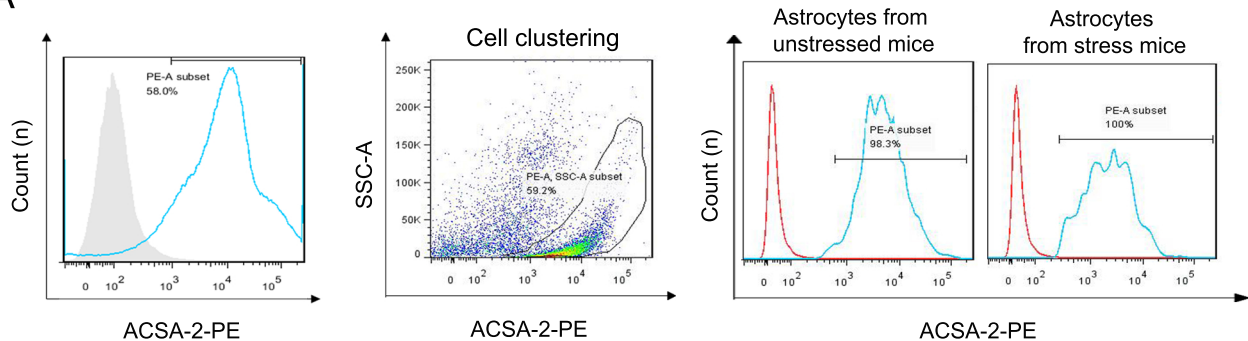

B

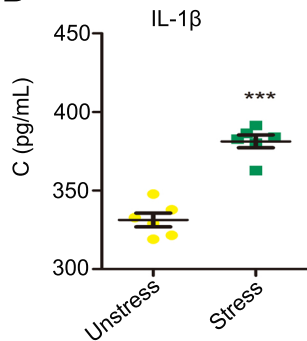

C

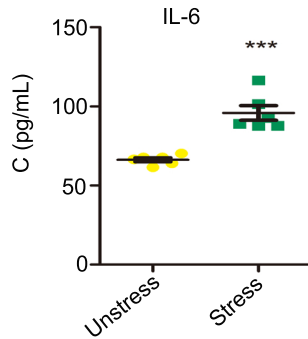

D

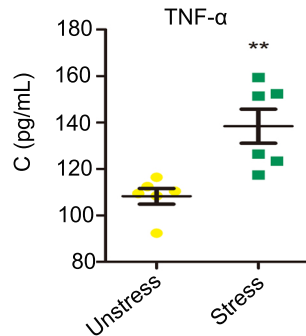

Supplement: Supplementary file 3 — Additional file 3: Figure S3. Extraction of astrocytes and ELISA analysis of their expression of inflammatory factors. (A) Extraction of astrocytes from stressed mice and unstressed mice by ACSA-2+ sorting with flow cytometry. Both groups of cells had a purity in excess of 90%. (B) IL-1β concentration in astrocyte culture medium (***P<0.001 compared with the unstressed group. n=6). (C) IL-6 concentration in astrocyte culture medium (***P<0.001 compared with the unstressed group. n=6). (D) TNF-α concentration in astrocyte culture medium (**P<0.01 compared with the unstressed group. n=6). [file 12974_2020_1787_MOESM3_ESM.pdf]

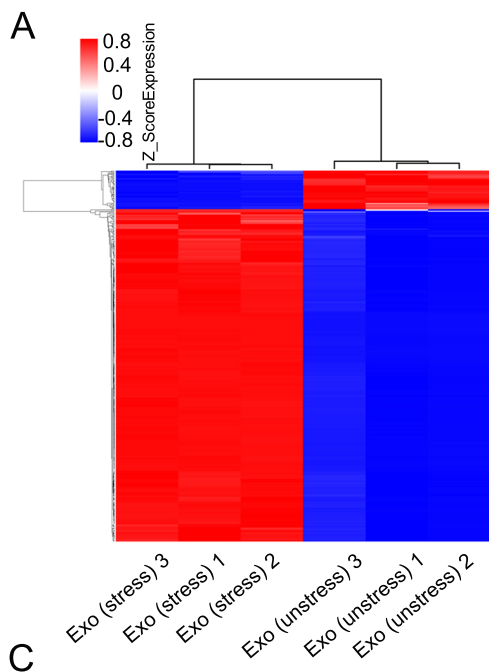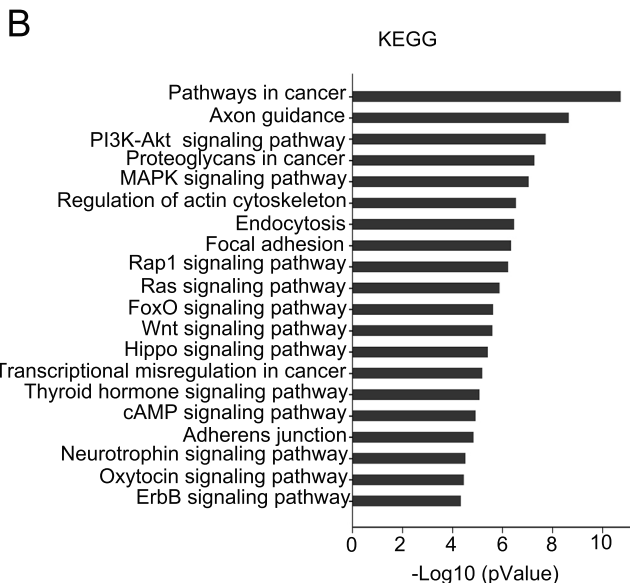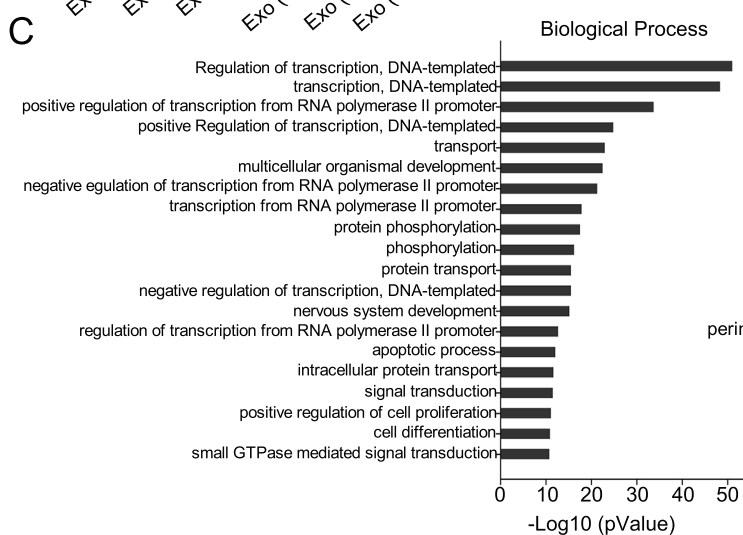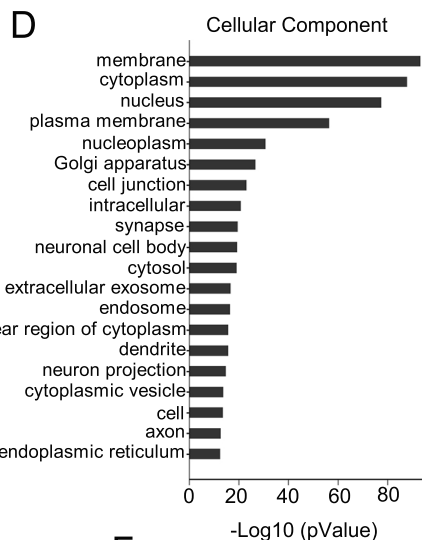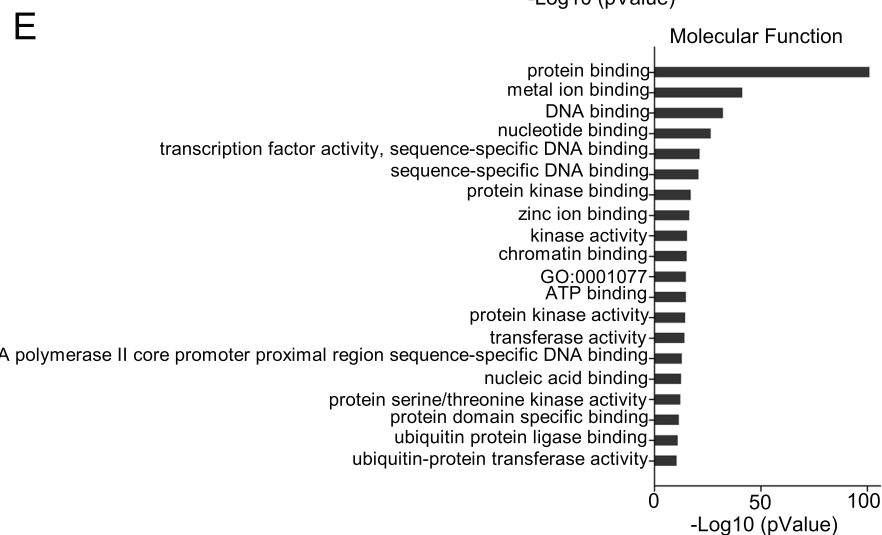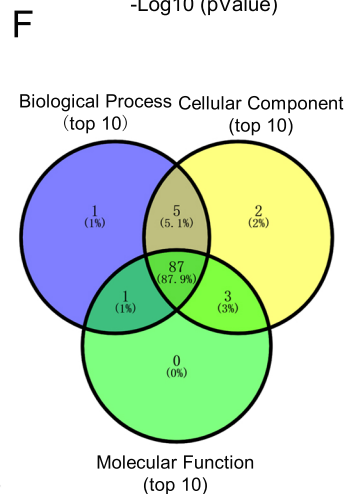

Supplement: Supplementary file 4 — Additional file 4: Figure S4. Bioinformatics analysis of miRNA arrays. (A) Heatmap of miRNAs between exo (unstress) exosomes from unstressed mouse NK cells and exo (stress) exosomes from stressed mouse NK cells. (B) Top 20 KEGG pathway analysis of exo (unstress) compared with exo (stress). (C) Top 20 biological process analyses of exo (unstress) compared with exo (stress). (D) Top 20 cellular component analysis of exo (unstress) compared with exo (stress). (E) Top 20 molecular function analyses of exo (unstress) compared with exo (stress). (F) Venn diagram of the top 10 biological process analyses, top 10 cellular component analyses and top 10 molecular function analyses. [file 12974_2020_1787_MOESM4_ESM.pdf]

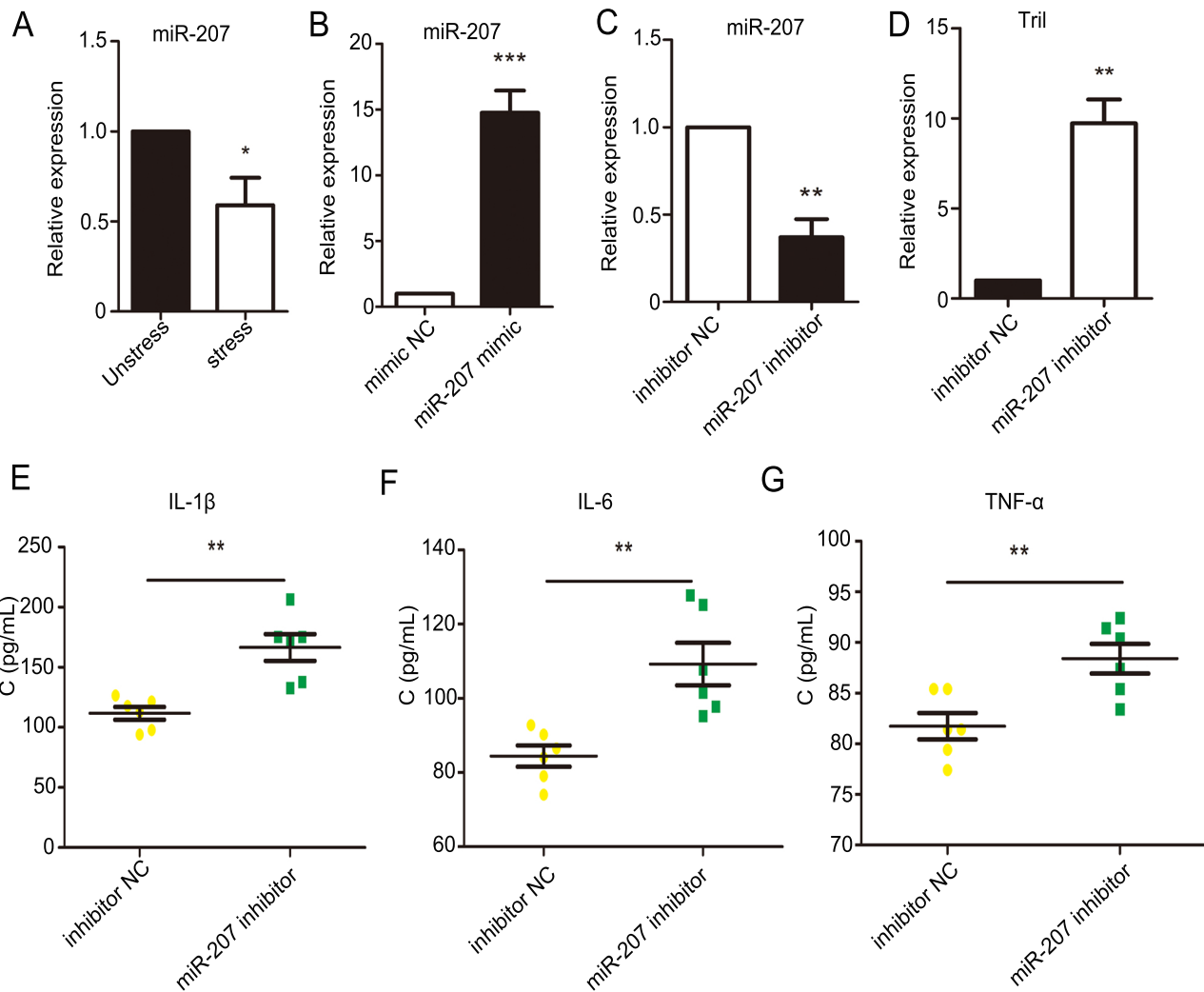

Supplement: Supplementary file 5 — Additional file 5: Figure S5. Expression of miR-207 in astrocytes and the functions of the miR-207 inhibitor in astrocytes in vitro. (A) Relative expression of miR-207 in astrocytes compared with the unstressed group (*P<0.05 compared with the unstressed group. n=6). (B) The relative expression of miR-207 in astrocytes after transfection with miR-207 mimic compared with transfection with a mimic NC (***P<0.001 compared with the mimic NC group. n=6). (C) The relative expression of miR-207 in astrocytes after transfection with miR-207 inhibitor compared with transfection with an inhibitor NC (**P<0.01 compared with the inhibitor NC group. n=6). (D) qPCR analysis of Tril mRNA expression after transfecting astrocytes with miR-207 inhibitor (**P<0.01 compared with the inhibitor NC group. n=6). (E) ELISA analysis of IL-1β concentration after transfecting astrocytes with miR-207 inhibitor (**P<0.01 compared with the inhibitor NC group. n=6). (F) ELISA analysis of IL-6 concentration after transfecting astrocytes with miR-207 inhibitor (**P<0.01 compared with the inhibitor NC group. n=6). (G) ELISA analysis of TNF-α concentration after transfecting astrocytes with miR-207 inhibitor (**P<0.01 compared with the inhibitor NC group. n=6). [file 12974_2020_1787_MOESM5_ESM.pdf]

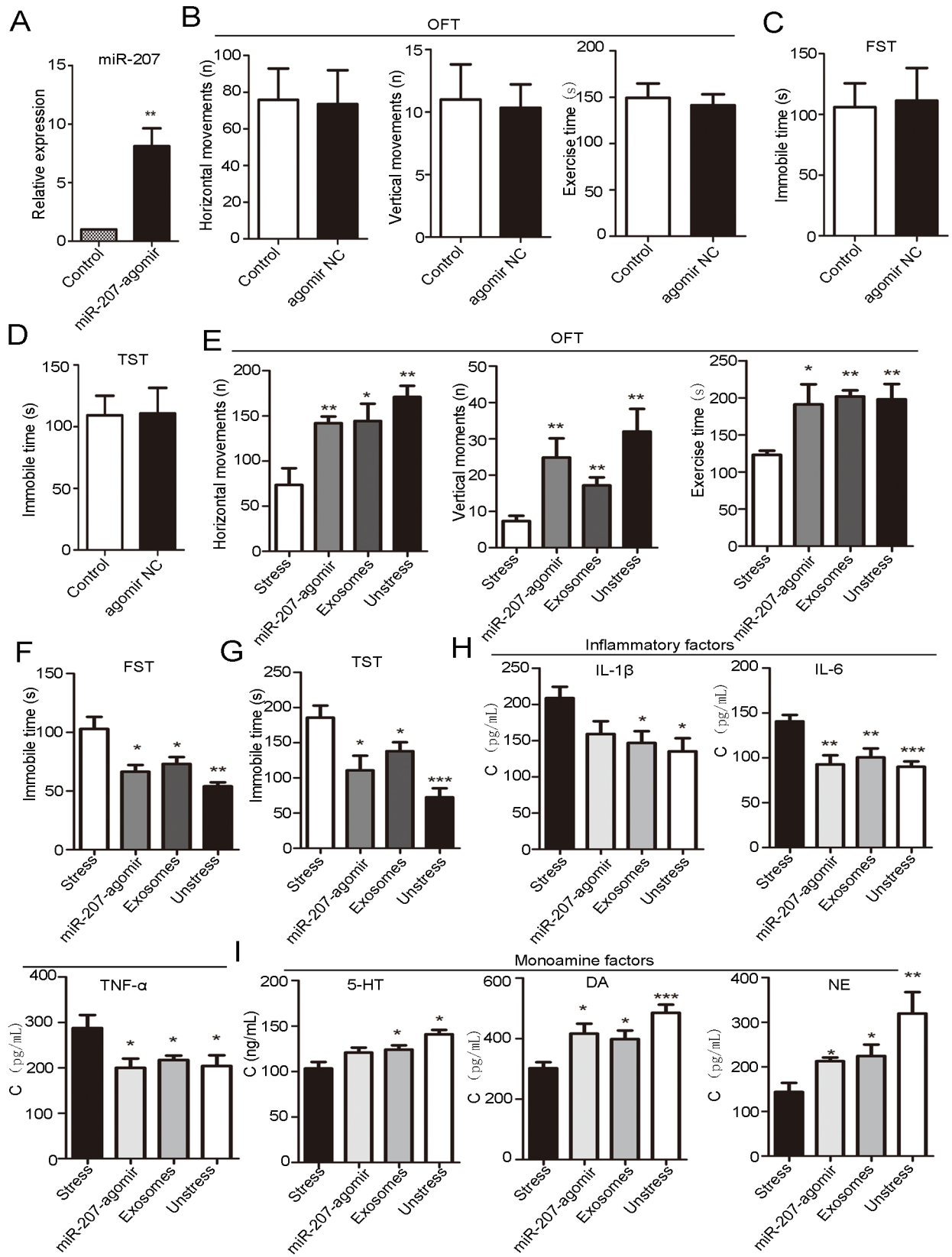

Supplement: Supplementary file 6 — Additional file 6: Figure S6. In vivo antidepressant activity of miR-207. (A) Relative expression of miR-207 in the hippocampus after intracranial injection of miR-207 agomir (**P < 0.01 compared with the control group. n=4). (B-D) Behavioral test after injection of agomir NC to evaluate the influence of the injection procedure. (B) Behavioral test (OFT), which included horizontal movements (defined as at least three paws in a square), vertical movements (defined as at least three paws in a square) and exercise time, which were compared with the results from the control group. (C) Behavioral test (FST) (recorded immobile time in seconds) results were compared with those of the control group. (D) Behavioral test (TST) (immobility was defined as hanging in a downright direction with only small movements) results were compared with those of the control group. (E-I) In vivo antidepressant activity of miR-207. The animal grouping strategy is shown in Table 3. (E) Behavioral (OFT), which included horizontal movements (defined as at least three claws in a square), vertical movements (defined as at least three claws in a square) and exercise time (*P<0.05, **P<0.01 compared with the stress group. n=6 in each group). (F) Behavioral test (FST) (recorded immobile time in seconds) (*P<0.05, **P<0.01 compared with the stress group. n=6 in each group). (G) Behavioral test (TST) (immobility was defined as hanging in a downright direction with only small movements) (*P<0.05, and ***P<0.001 compared with the stress group. n=6 in each group). (H) ELISA analysis of inflammatory factor concentrations, IL-6, IL-1β, and TNF-α, in the brain (*P<0.05, **P<0.01, and ***P<0.001 compared with the stress group. n=6 in each group). (I) ELISA analysis of monoamine factor concentrations, 5-HT, DA and NE, in the brain (*P<0.05, **P<0.01, and ***P<0.001 compared with the stress group. n=6 in each group). [file 12974_2020_1787_MOESM6_ESM.pdf]
